# Genome-Wide Identification of Autophagy-Related Gene Family and Gene Expression Analysis of the *CmATG8* Under Heat Stress in *Chrysanthemum*

**DOI:** 10.3390/ijms26178642

**Published:** 2025-09-05

**Authors:** Bing-Yu Luo, Hui Meng, Zheng-Yu Lu, Peng Wang, Cheng-Shu Zheng, Xiao-Yun Wu, Dong-Ru Kang, Wen-Li Wang

**Affiliations:** College of Horticultural Science and Engineering, Shandong Agricultural University, Tai’an 271018, China; luoyyyy610@163.com (B.-Y.L.); menghuiii98@163.com (H.M.); lzy15963402751@163.com (Z.-Y.L.); kanyun2000@126.com (P.W.); zcs@sdau.edu.cn (C.-S.Z.); xiaoyunwu@sdau.edu.cn (X.-Y.W.)

**Keywords:** *Chrysanthemum*, *CmATG8*, high temperature, expression analysis, gene family

## Abstract

*Chrysanthemum morifolium* is one of the world’s four major cut flowers, valued for its ornamental and economic importance. However, high temperature stress during growth and development can reduce both yield and quality. Autophagy is a cellular self-degradation and recycling process that plays a pivotal role in maintaining homeostasis under abiotic stress. This study aimed to identify autophagy-related genes (*ATGs*) in *C*. *morifolium* and its close relatives, analyze their structural and evolutionary characteristics, and evaluate *ATG8* expression under heat stress. Genome-wide analysis identified 130 *ATGs* in *C*. *morifolium*, 51 in *Chrysanthemum nankingense*, and 49 in *Chrysanthemum lavandulifolium*. Genes within the same subfamily exhibited conserved structures and domains, with fragment duplication contributing to *ATG* expansion. Expression profiling showed that *ATG8* genes were the most highly expressed and displayed tissue specificity, while heat stress induced their transcription, peaking at 48 h. These findings provide a comprehensive genomic resource for *Chrysanthemum ATGs* and indicate a potential role for *ATG8* in heat stress responses, offering a basis for future studies aimed at improving thermotolerance in this ornamental crop.

## 1. Introduction

In plants, autophagy occurs in three main forms [1]. Macroautophagy forms double-membrane autophagosomes around cellular components, which fuse with vacuoles or lysosomes for degradation and recycling [2]. Microautophagy involves direct invagination of the vacuolar or lysosomal membrane to engulf cytoplasmic components without forming double-membrane autophagosomes [3]. Mega autophagy occurs under programmed cell death or severe stress, where vacuolar rupture releases hydrolytic enzymes to rapidly degrade cellular contents [4]. In plants, autophagy is maintained at low basal levels under normal conditions but is rapidly activated by abiotic stresses such as nutrient deprivation, drought, salinity, and heat [5,6,7,8,9].

Accumulating evidence has demonstrated that the expression of *ATGs* plays a critical role in modulating plant adaptation to various environmental stresses. For instance, in *Arabidopsis thaliana*, mutants deficient in *ATG5*, *ATG7*, or *NBR1* exhibit compromised cellular membrane integrity and increased damage to photosystem II (PSII) under heat stress compared to wild-type plants. These mutants also accumulate higher levels of insoluble protein aggregates and display significantly lower survival rates under drought, salinity, and oxidative stress conditions [10]. In contrast, plants overexpressing *AtATG5* or *AtATG7* demonstrate enhanced autophagic activity, delayed senescence, prolonged flowering periods and lifespan, and increased resistance to the necrotrophic pathogen *Alternaria brassicicola*, as evidenced by suppressed fungal growth in transgenic lines [11]. Moreover, transgenic *Arabidopsis*, tobacco, and grapevine calli overexpressing *VvATG6*, generated via Agrobacterium-mediated transformation, exhibit significantly enhanced antioxidant enzyme activities following copper (Cu) exposure. These lines also show reduced accumulation of malondialdehyde (MDA) and hydrogen peroxide (H_2_O_2_), along with downregulated expression of ROS-generating genes RbohB and RbohC, thereby improving Cu stress tolerance [12]. In *Solanum lycopersicum*, the heat shock transcription factor *HsfA1* plays a key role in drought tolerance. Research has shown that *SlHsfA1a* binds to the promoter regions of *ATG10* and *ATG18f*, suggesting that it enhances autophagy by regulating *ATG* gene expression, thereby increasing the plant’s ability to withstand drought stress [13].

Under abiotic stress conditions, plants often experience an accumulation of misfolded or unfolded proteins within the endoplasmic reticulum (ER), thereby triggering the ER stress response [14]. Autophagy, a major intracellular degradation pathway for macromolecules, has been shown to be activated by ER stress through the ER stress sensor INOSITOL-REQUIRING ENZYME 1b (IRE1b), which facilitates the sequestration and transport of ER fragments to the vacuole for degradation. Notably, heat stress can induce ER stress, thereby promoting autophagy activation [15]. *ATG8* is essential for autophagosome formation, selective autophagy execution, and cargo recognition. Through interactions with specific autophagy receptors, *ATG8* mediates the targeted degradation of damaged organelles and protein aggregates [16]. Studies on the *A. thaliana ATG8* gene family have shown its involvement in plant growth, development, and stress adaptation [17,18,19]. *ATG8* also contributes to the recovery of the Golgi apparatus following heat stress [20,21] and directly interacts with the selective autophagy receptor *NBR1*. In *A. thaliana*, mutants lacking *NBR1* accumulate high levels of insoluble proteins under heat stress, highlighting its role in maintaining protein homeostasis [22]. In *Capsicum annuum*, silencing the *CaATG8c* gene reduces chlorophyll levels and increases malondialdehyde content under heat stress, ultimately lowering the plant’s heat tolerance [23]. In contrast, overexpressing *SlATG8f* in *S. lycopersicum* weakens the plant’s overall heat tolerance but significantly improves pollen viability and reduces damage to chloroplasts and mitochondria under high temperatures [24]. These findings suggest that *ATG8* is not only a central regulator of autophagy but also plays a vital role in helping plants cope with heat stress.

With its rich colors, diverse flower forms, and extended blooming period, *Chrysanthemum* is widely used in landscape design, potted displays, and the cut flower market. However, under the influence of global climate change, high temperature stress has become one of the major limiting factors affecting its growth, development, and industrial production [25]. In *Chrysanthemum* cultivation, the optimal growth temperature is around 25 °C; when ambient temperatures exceed this threshold, plant growth and development slow markedly, and at 40 °C, growth nearly ceases. During the vegetative growth stage, chrysanthemums frequently encounter high summer temperatures, resulting in growth arrest, delayed flowering, and reduced flower coloration, all of which are key factors in limiting flowering quality and yield [26]. Therefore, enhancing thermotolerance in *Chrysanthemum* has become an urgent priority for ensuring the sustainability of the industry. Autophagy is a critical cellular process in plant stress responses; however, the *ATG* gene family remains largely uncharacterized in *Chrysanthemum* and its relatives. This study presents a comprehensive bioinformatic and tissue-specific expression analysis of *ATG* genes, with a focus on their transcriptional responses to heat stress. The results provide essential genetic resources and a basis for the molecular breeding of a heat-tolerant chrysanthemum via genetic engineering.

## 2. Results

### 2.1. Gene Family Identification and Physicochemical Property Analysis

A total of 130, 49, and 51 *ATG* gene family members were identified in *C. morifolium*, *C. nankingense*, and *C. lavandulifolium*, respectively. Within each subfamily, genes were named sequentially according to their sequence ID order. Based on protein domain features, the *CmATG* gene family is classified into 22 subfamilies (Supplementary Table S1). The length of the proteins encoded by the *CmATG* family ranges from 86 (CmATG10e) to 2464 amino acids (CmTORd), with molecular weights spanning from 9847.33 Da (CmATG10e) to 276,398.94 Da (CmTORd). The predicted results show that the isoelectric points of CmATG proteins range from 4.58 (CmATG3a) to 9.63 (CmATG12c), the instability index ranges from 30.58 (CmATG8b) to 76.48 (CmATG13a), and the aliphatic index varies between 65.33 (CmATG13a) and 106.59 (CmATG7d). The grand average of hydrophilicity is negative for all but nine *CmATGs*, suggesting that the majority of CmATG proteins are hydrophilic.

The *CnATG* and *ClATG* gene families contain 21 subfamilies each (Supplementary Tables S2 and S3). The proteins encoded by the *CnATG* and *ClATG* family range in length from 96 (CnATG12) to 2507 amino acids (CnTORa), and from 96 (ClATG12) to 2464 amino acids (ClTORb), with molecular weights ranging from 10,664.2 Da (CnATG12) to 281,609.62 Da (CnTORa), and from 10,567.09 Da (ClATG12) to 276,458.09 Da (ClTORb). The isoelectric points, instability indices, and aliphatic indices of the CnATG and ClATG proteins are similar to those of the CmATG proteins, with the grand average of hydrophilicity values all being negative.

### 2.2. Phylogenetic Analysis

**Figure 1:**
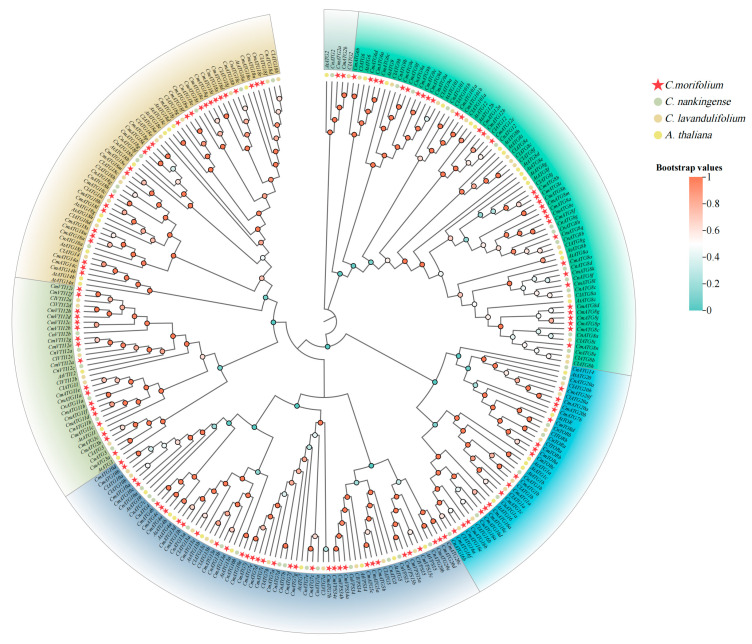
Phylogenetic analysis of *CmATGs*, *ClATGs*, *CnATGs,* and *AtATGs.* The phylogenetic tree of 130 *CmATGs*, 49 *CnATGs*, 51 *ClATGs*, and 45 *AtATGs* was constructed using the Neighbor-Joining method in MEGA11, with the bootstrap test set to 1000 replicates. Red pentagrams represent *CmATG*, green circles indicate *CnATG*, orange circles denote *ClATG*, and yellow circles correspond to *AtATG* genes. The green to orange color represents the low to high bootstrap value.

To explore the evolutionary relationships among *ATG* genes, a phylogenetic tree was constructed in MEGA 11.0 based on 130 CmATG protein sequences, together with 45 AtATG proteins and the identified 49 and 51 ATG proteins from *C. nankingense* and *C. lavandulifolium*, respectively. The phylogenetic analysis (Figure 1) classified the ATG proteins into six major groups. Most homologous genes clustered together within the same subgroup, except for members of the *ATG7*, *ATG14*, and *ATG20* subfamilies, which were distributed across different branches. The evolutionary relationships among species varied between subfamilies; however, compared to *A. thaliana*, *C. morifolium ATG* proteins exhibited a closer evolutionary relationship with those of *C. nankingense* and *C. lavandulifolium*.

### 2.3. Gene Structure, Conserved Motif, and Domain Analysis of the ATG Gene Family

**Figure 2:**
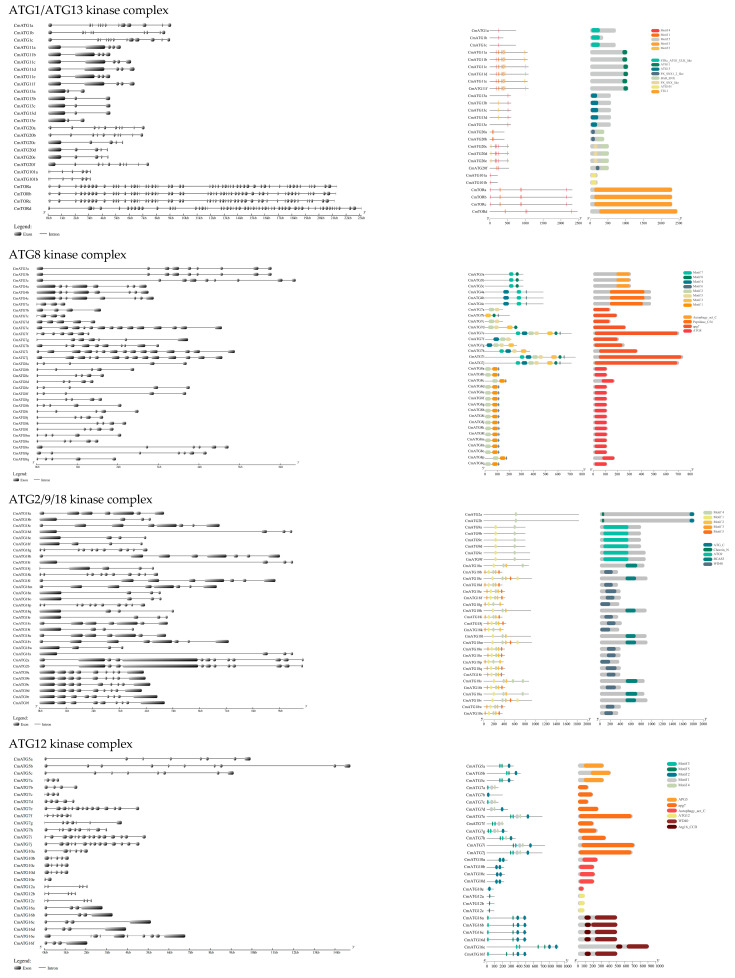

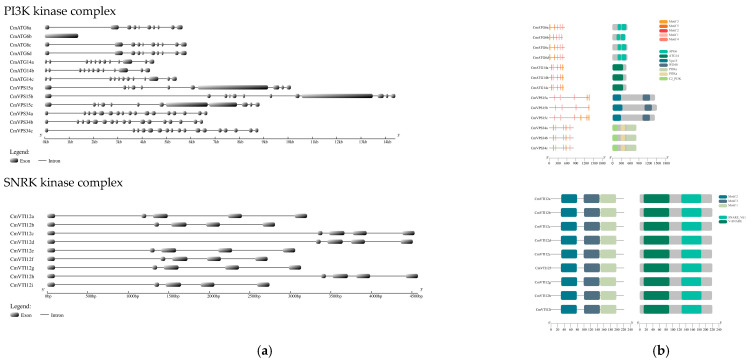
Gene structures, domains, and motifs of CmATGs. The 130 *CmATG* genes were presented according to their functions in autophagy. (**a**) The exon–intron structures, with exons in black rectangles and introns in thin black lines. (**b**) Domains and motif of the corresponding genes on the right side. The 130 CmATGs were classified based on their association with autophagy-related protein kinase complexes. Genes within the same subfamily exhibited similar exon–intron structures, conserved domains, and motif compositions.

The *CmATG* genes were classified based on their associated protein kinase complexes, and their gene structures, conserved domains, and motifs were analyzed and visualized. The results (Figure 2) show that genes within the same subfamily exhibit specific characteristics in terms of gene structure, conserved domains, and motif composition. The number of introns in the *CmATG* gene family ranged from 0 to 55, while the number of exons varied from 1 to 54. For example, *CmATG6b* has no introns and only one exon, whereas *CmTORb* contains 55 introns and 54 exons. In the *CnATG* genes, intron numbers ranged from 2 (*CnATG10b*) to 58 (*CnTORa*), and in the *ClATG* genes, intron numbers ranged from 0 (*ClATG6*) to 56 (*ClTORa* and *ClTORb*) (Supplementary Figures S1 and S2).

Motif analysis of the ATG proteins using MEME revealed that motifs varied across *CmATG* subfamilies, with similar motif patterns and quantities within the same subfamily (Figure 2). When analyzing all *CnATG* and *ClATG* subfamilies together, we identified 25 distinct motifs, though some members were missing certain motifs (Supplementary Figures S3 and S4). This suggests that the structural differences between ATG proteins across subfamilies make it more meaningful to analyze motifs according to their associated protein kinase complexes.

In terms of conserved domains, all CmATG proteins within the same subfamily shared the same conserved domains, except for CmATG18 (Figure 2). In the *CmATG18* subfamily, six members contained both BCAS3 and WD40 domains, while 17 members had only the WD40 domain. *CmATG18h* had only the BCAS3 domain. Similar patterns were observed in the *ClATG18* and *CnATG18* subfamilies (Supplementary Figures S3 and S4), suggesting functional diversity among these members.

### 2.4. Chromosomal Localization and Synteny Analysis of ATG Gene Family Members

**Figure 3:**
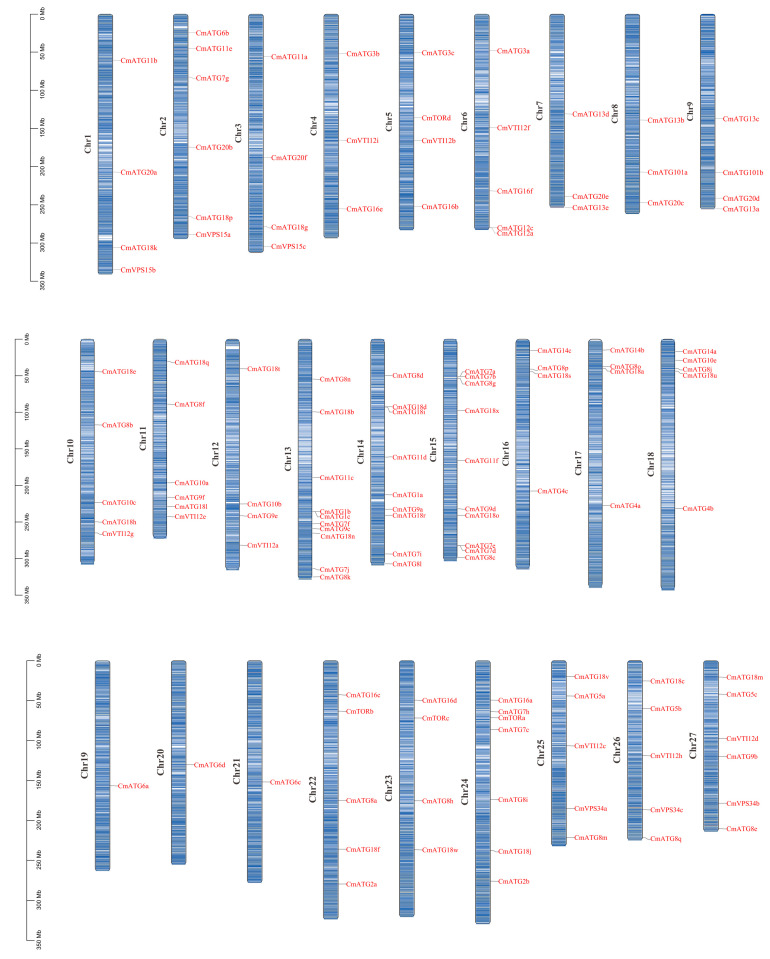
Chromosomal distribution of *CmATGs* in *C. morifolium*. A total of 130 *CmATGs* were mapped onto the 27 chromosomes. The gene names are labeled in red. The scale on the left represents chromosome length in megabases (Mb). The gene positions were determined based on the reference genome annotation.

Chromosomal localization analysis of *Chrysanthemum* showed that *CmATG10d* and *CmATG12b* have not yet been assigned to chromosomes. The remaining *CmATG* genes are distributed unevenly across the 27 chromosomes of *Chrysanthemum*. Chromosomes Chr13 and Chr15 each contain up to 10 *CmATG* genes, while chromosomes Chr19, Chr20, and Chr21 have the fewest, with only one *CmATG* gene each (Figure 3). The *CnATG* gene family has six members (*CnATG7d*, *CnATG7f*, *CnATG8f*, *CnATG10a*, *CnVTI12a*, *CnVTI12c*) that have not yet been assigned to chromosomes. Additionally, chromosome Chr3A in *C. nankingense* and LG03 in *C. lavandulifolium* lacked any *ATG* gene family members (Supplementary Figures S5 and S6).

**Figure 4:**
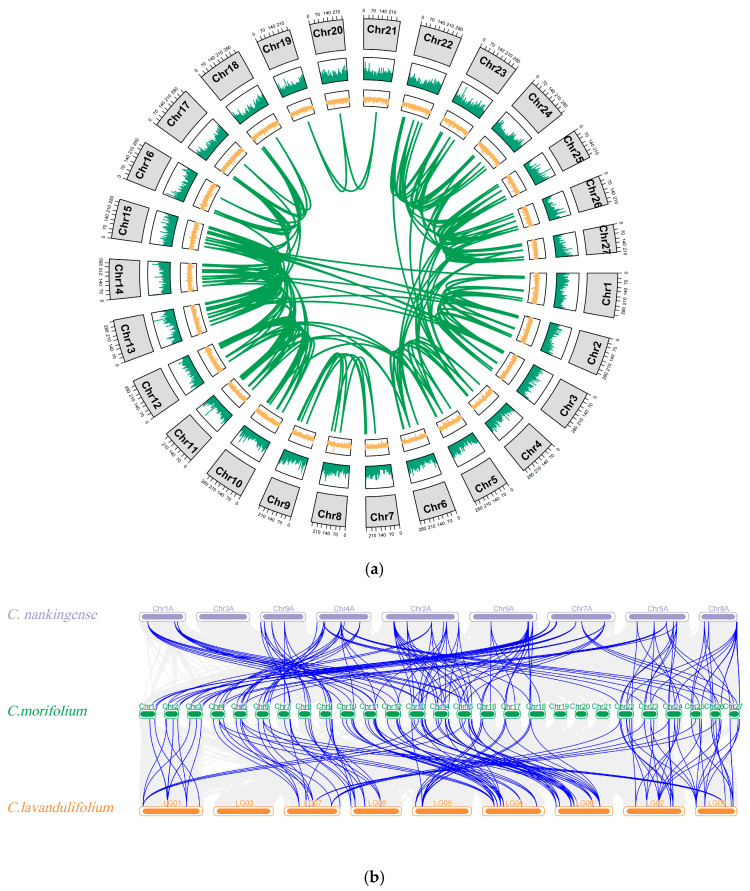
Collinearity analysis of *ATG* gene family. (**a**) Replication relationship of *CmATGs* in the *Chrysanthemum* genome. The outermost circle represents the 27 chromosomes of *C. morifolium* (Chr1–Chr27), labeled in black. The orange regions indicate gene density. Green lines in the center connect segmentally duplicated *CmATG* gene pairs, illustrating potential collinear relationships across the genome. The figure was generated using MCScanX and visualized with TBtools-II;. (**b**) Collinearity analysis of *CmATG*, *ClATG,* and *CnATG*. The green bars in the middle represent the chromosomes of *C. morifolium*, while the top and bottom bars indicate the pseudochromosomes or linkage groups of *C. nankingense* (Chr1A–Chr8A, top) and *C. lavandulifolium* (LG01–LG08, bottom), respectively. Blue lines represent syntenic gene pairs among the three species, highlighting conserved genomic regions and evolutionary relationships within the ATG gene family. The gray lines represent all syntenic relationships among the three *Chrysanthemum* genomes.

An intra-species synteny analysis of the *CmATG* gene family was performed, followed by an inter-species synteny analysis comparing *C. nankingense* and *C. lavandulifolium*. The results of the intra-species synteny analysis showed that the *CmATG* gene family does not contain tandem repeats but rather expands through segmental duplications, forming 252 pairs of syntenic genes (Figure 4a). The inter-species synteny analysis revealed 149 and 196 pairs of syntenic genes between *CmATG* and *CnATG*, and between *CmATG* and *ClATG*. Typically, three *CmATG* genes correspond to one *CnATG* and *ClATG* (Figure 4b).

### 2.5. Analysis of Cis-Regulatory Elements in the Promoters of the Chrysanthemum ATG Gene Family

**Figure 5:**
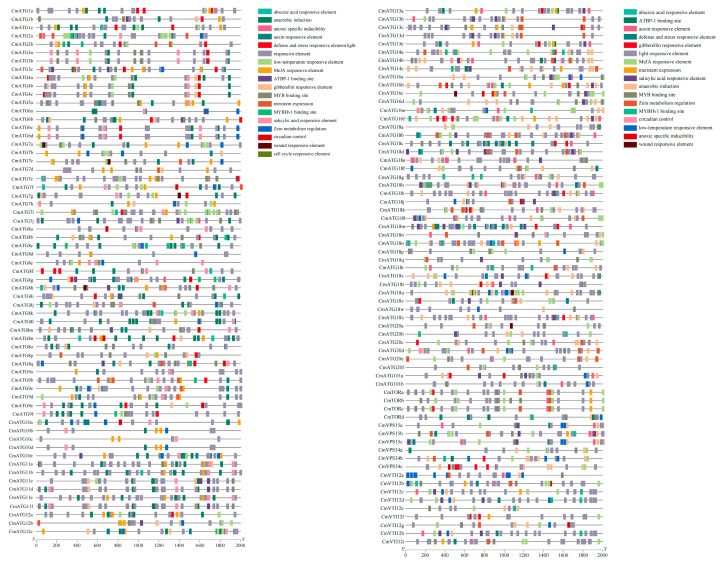
Analysis of cis-acting regulatory elements in the promoter regions of *CmATG* genes. The distribution and abundance of different cis-regulatory element types are shown for each gene. Promoter sequences comprising 2000 bp upstream of the predicted translation start site (ATG) were retrieved and analyzed using PlantCARE.

Cis-regulatory element analysis revealed 18 common cis-regulatory elements in the *Chrysanthemum ATG* gene family, which were classified into five categories (Figure 5, Supplementary Figures S7 and S8). These include elements associated with abiotic stress (five elements), plant hormone responses (seven elements), plant growth and development (two elements), light response, and transcription factor binding sites (three elements). The *CmATG* genes contain diverse cis-regulatory elements linked to abiotic stress responses, including ARE (anaerobic response element), MBS (MYB-binding site involved in drought response), and LTR (low-temperature responsiveness), as well as hormone-responsive elements such as ABRE (ABA-responsive element), TGA (auxin-responsive element), and CGTCA motifs (MeJA-responsive element). These elements suggest a significant role for *CmATG* genes in mediating stress adaptation and hormonal regulation in chrysanthemum. Furthermore, 29 members of the *CmATG* gene family harbor growth- and development-related elements, such as CAT-box (meristem expression) and O2-site (zein metabolism regulation), indicating their potential involvement in developmental processes. Notably, 105 *CmATG* genes possess binding sites for key transcription factors, including MYB, MYBHv1, and ATBP-1, underscoring their regulatory complexity and potential involvement in transcriptional control under various physiological and stress conditions. These results suggest that *CmATG* genes may play a pivotal role in coordinating chrysanthemum responses to abiotic stresses and hormone-mediated regulatory networks, contributing to its stress resilience and developmental regulation.

### 2.6. Tissue-Specific Expression of the Chrysanthemum ATG Gene Family

To confirm whether the identified *CmATG* genes are normally expressed in Chrysanthemum, the expression of 130 *ATG* gene members in different tissues was analyzed. Expression data for the *ATG* genes were obtained from the *Chrysanthemum* transcriptome database [27]. The transcription levels of all members were compiled (Supplementary Table S4). Among these, 111 genes showed differential expression across different tissues, while the remaining 19 genes had FPKM values close to 0 or 0 in all tissues. FPKM (fragments per kilobase of transcript per million mapped reads) was used to quantify transcript levels; this metric normalizes read counts for both transcript length and sequencing depth, and values near zero indicate negligible or no detectable expression.

**Figure 6:**
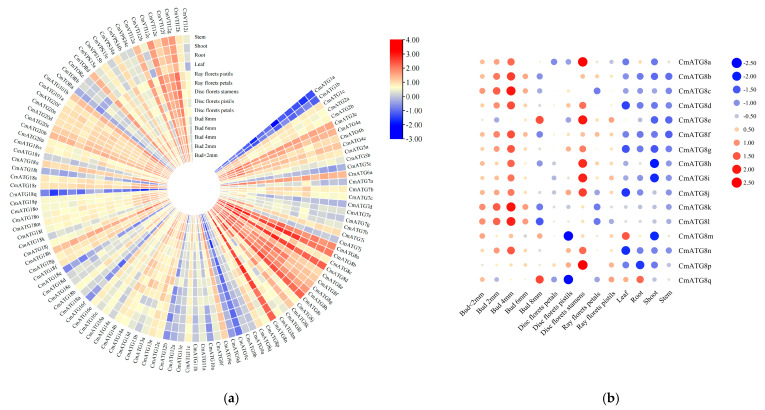
*CmATG* gene expression in different tissue sites. (**a**) Circular heatmap illustrating the transcriptional levels of *CmATG* genes across various tissues. The expression levels are indicated by a color scale, with red representing higher transcript abundance and blue representing lower expression levels. (**b**) Bubble chart showing the tissue-specific expression patterns of genes from the *CmATG8* subfamily. The size of each bubble corresponds to the expression magnitude, while the color intensity reflects relative expression levels across tissues.

The expression levels of all detected genes were analyzed and visualized (Figure 6a). The results revealed significant variation in the expression levels of different *CmATG* family members. Genes from the *CmATG8* and *CmVTI12* subfamilies generally exhibited higher expression levels, while genes from other subfamilies, such as *CmATG1a*, *CmATG1b*, and *CmATG9c*, showed lower expression levels. Analysis of the expression in various tissues showed that *CmATG8b* was prominently expressed in tubular flower petals, ray florets, pistils of ray florets, and shoot apical meristems. In tubular flower pistils, *CmATG8n* had the highest expression, while *CmATG8d* was most highly expressed in tubular flower stamens, roots, apical tissues, and stems. In leaves, *CmATG8i* was the most highly expressed gene.

A differential expression analysis of the 18 *CmATG8* family genes across various tissues revealed that *CmATG8s* showed a trend of increasing expression followed by a decrease during different stages of flower bud development. The highest transcription levels were observed in 4 mm flower buds, suggesting that autophagy activity is higher during the early stages of flower bud development under normal growth conditions (Figure 6b). This implies that the *CmATG8* subfamily plays a role in the differentiation and formation of flower buds.

### 2.7. Expression Analysis of CmATG8 Subfamily in Response to Heat Stress

**Figure 7:**
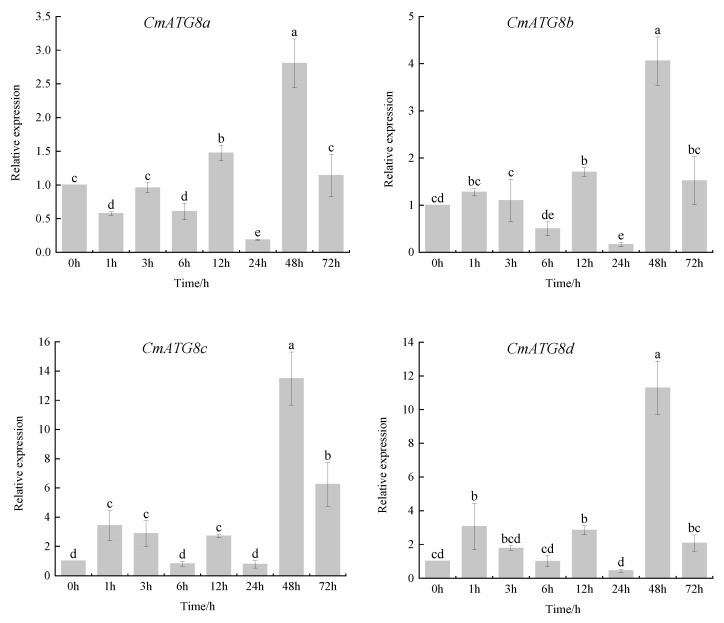

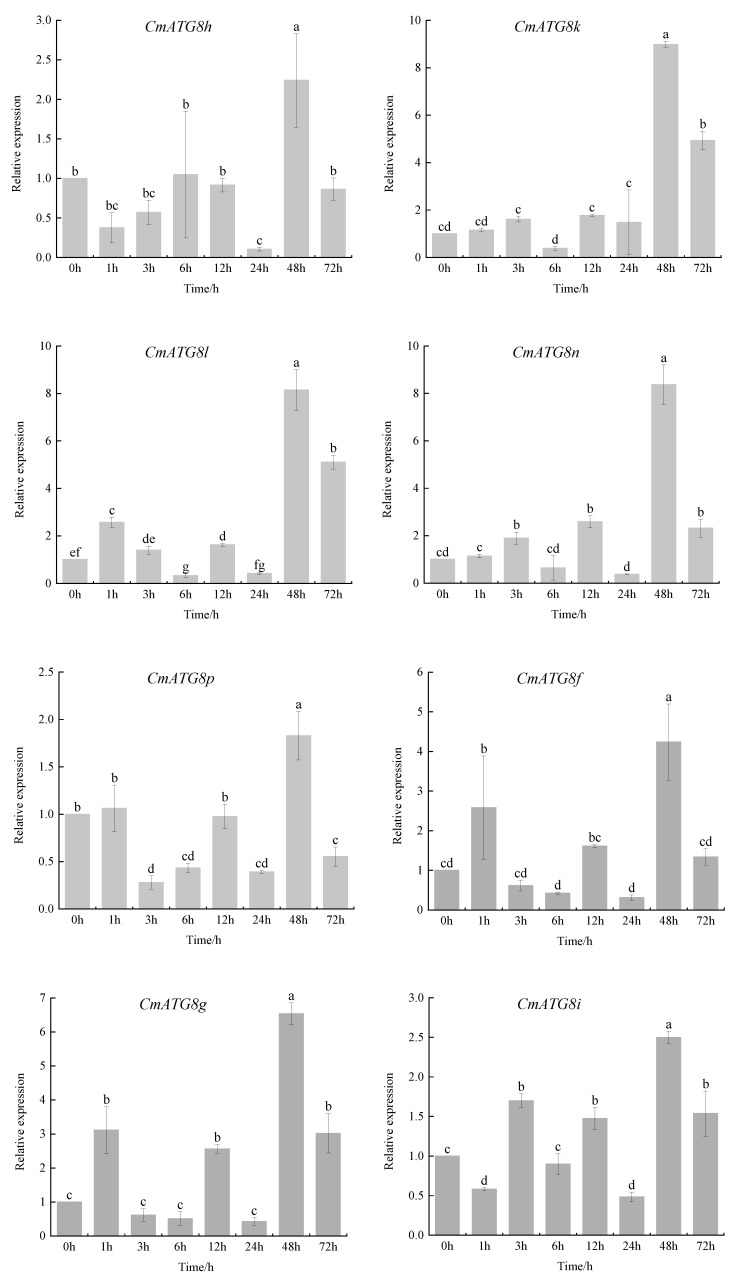

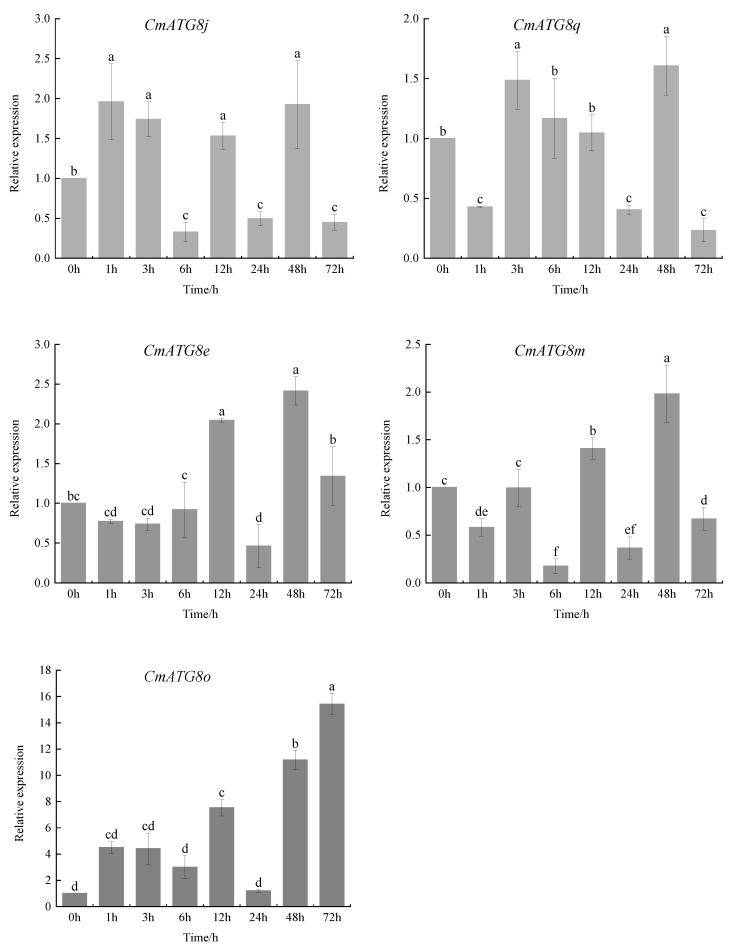
Expression profiles of 17 *CmATG8* genes under heat stress as determined by qRT-PCR. Leaves of *Chrysanthemum* were collected at eight time points (0, 1, 3, 6, 12, 24, 48, and 72 h) after heat treatment to assess the relative expression levels of *CmATG8* genes. Gene expression was normalized to the internal reference gene *CmEF1α*, and relative quantification was performed using the 2^−ΔΔCt^ method. Data represent the mean ± standard deviation (SD) of three biological replicates. Statistical significance was assessed using one-way ANOVA followed by Duncan’s multiple range test (*p* < 0.05); different lowercase letters indicate significant differences among time points for each gene.

The *CmATG8* gene family exhibited relatively high transcript levels across various tissues, suggesting its potential involvement in stress-related processes. To investigate their roles in heat stress response, the expression dynamics of *CmATG8* genes were examined by qRT-PCR in chrysanthemum leaves sampled at eight time points within a 72 h period following heat treatment. As shown in Figure 7, all *CmATG8* members responded to heat stress, though with distinct temporal expression patterns that could be grouped into four major categories.

*CmATG8a*, *CmATG8b*, *CmATG8c*, *CmATG8d*, *CmATG8h*, *CmATG8k*, *CmATG8l*, *CmATG8n*, and *CmATG8p* displayed low expression during the initial 24 h of treatment, followed by a pronounced upregulation at 48 h and a subsequent decline by 72 h. Among these, *CmATG8c* and *CmATG8d* showed the most substantial increases, with transcript levels rising by 13.4-fold and 11.2-fold, respectively, while *CmATG8k* and *CmATG8l* increased by 8.9-fold and 8.1-fold, respectively. *CmATG8f*, *CmATG8g*, *CmATG8i*, *CmATG8j*, and *CmATG8q* exhibited a rapid early response, with peak expression observed at 1 h or 3 h post-treatment. Notably, *CmATG8g* expression increased by 3.1-fold at 1 h. *CmATG8e* and *CmATG8m* showed a distinct pattern of response; their expression levels began to increase at 12 h, initially declining from 12 to 24 h before rising again from 24 to 48 h. Most *CmATG8* genes reached their lowest expression levels at 24 h of treatment. Interestingly, *CmATG8o* exhibited a distinct and sustained upregulation: its transcript levels rose approximately 4.5-fold at 1 h and 3 h, peaked at a 7.5-fold increase by 12 h, declined slightly at 24 h, and then continued to rise between 48 h and 72 h, ultimately reaching a 15.4-fold increase relative to the untreated control. These findings suggest that *CmATG8o* may play a critical role in mediating *Chrysanthemum*’*s* response to heat stress.

## 3. Discussion

Autophagy is an essential process in plant growth and development, playing a vital role in maintaining cellular homeostasis, delaying senescence, and enhancing stress tolerance. This complex biological mechanism is orchestrated by a series of ATG proteins with distinct functions. To date, the *ATG* gene family has been identified and analyzed in multiple plant species, including *Arabidopsis thaliana* (39 genes) [28], *Oryza sativa* (33 genes) [29], *Camellia sinensis* (80 genes) [30], *Triticum aestivum* (108 genes) [31], *Medicago truncatula* (39 genes) [32], *Nicotiana tabacum* (30 genes) [33], *Fagopyrum tataricum* (49 genes) [34], Beta vulgaris (29 genes) [35], *Lycium ruthenicum* (36 genes) [36], and *Solanum tuberosum* (29 genes) [37].

Based on whole-genome data, 130 *ATG* genes were identified in *C. morifolium*, 49 in *C. nankingense*, and 51 in *C. lavandulifolium*. The observed gene numbers aligned with the species’ ploidy levels, indicating a possible correlation between gene family expansion and genome duplication. Gene copy number analysis showed that the expansion of the *CmATG8* subfamily is independent of ploidy level. *C. morifolium* contains 17 *ATG8* genes, while *C. nankingense* and *C. lavandulifolium* contain eight and nine copies. ATG8 is crucial for autophagosome membrane formation via covalent binding to phosphatidylethanolamine (PE), and its essential role under environmental stresses likely subjects it to strong purifying selection, preserving stable copy numbers regardless of ploidy [38]. Similar patterns have been reported in wheat, where *ATG8* expansion is unaffected by species ploidy [39]. The comparatively larger *CmATG* gene family may be linked to the evolutionary history of *C. morifolium*. Previous studies indicate that chrysanthemums have undergone multiple whole-genome triplication (WGT) events, along with recent bursts of long terminal repeats (LTRs), resulting in a highly complex and expanded genome structure [27].

Previous studies identified 22 *ATG* subfamilies in *A. thaliana* [28] and 24 subfamilies in *C. sinensis* [30]. A total of 22, 21, and 21 *ATG* subfamilies were detected in *CmATG*, *CnATG*, and *ClATG*, respectively, with the ATG6 subfamily absent in *C. nankingense* and the ATG4 subfamily absent in *C. lavandulifolium*. ATG6 is a core component of the PI3K protein kinase complex, playing a critical role in autophagosome nucleation and vacuolar degradation [40]. On the other hand, ATG4 functions as a cysteine protease that cleaves the C-terminal region of ATG8, enabling it to execute its role in promoting autophagic vesicle formation and autophagosome maturation [18]. These differences suggest that the regulation of autophagy may vary among *C. morifolium*, *C. nankingense*, and *C. lavandulifolium*, potentially reflecting genomic divergence. This finding provides new insights into the evolution and functional adaptation of autophagy-related genes in *Asteraceae* species. Synteny analysis revealed 252 segmental duplication pairs among *CmATG* genes, with nearly one *CnATG* or *ClATG* gene corresponding to three *CmATG* genes, suggesting that *ATG* genes expanded through gene duplication during polyploidization while maintaining high structural conservation.

Cis-regulatory elements are fundamental to the transcriptional regulation of *ATG* family members. Different transcription factors (TFs) recognize specific cis-elements, enabling precise control over *ATG* gene expression, thereby influencing autophagic activity and stress resilience in plants. In *A. thaliana*, the TGA9 transcription factor, a member of the bZIP family, specifically binds to the TGACG (TGA) cis-element located in the promoters of *AtATG8B* and *AtATG8E*. The overexpression of *AtTGA9* significantly upregulates the expression of these two genes, promoting autophagosome formation and enhancing plant tolerance under nutrient deficiency and abiotic stress conditions such as high salinity and drought [17]. In Chinese flowering cabbage (*Brassica rapa* ssp. parachinensis), *BrMYB108*, an R2R3-MYB transcription factor, has been shown to directly interact with the promoters of *BrATG5b*, *BrATG8e-1*, and *BrATG8h-1*. Overexpression of *BrMYB108* not only activates autophagy under normal conditions but also enhances autophagic activity under sucrose starvation and osmotic stress, thereby improving plant stress tolerance [41]. WRKY transcription factors are also involved in regulating *ATG* gene expression by recognizing W-box elements (core sequence: TTGACC/T) within their promoters. For instance, *WRKY33* in *Arabidopsis* interacts with *ATG18a* during plant defense responses, conferring enhanced resistance against necrotrophic pathogens [42]. Similarly, its homologs *SlWRKY33a* and *SlWRKY33b* in tomato are implicated in heat stress responses, where they regulate autophagy-related gene expression to improve thermotolerance [43]. Additionally, the ethylene response factor *ERF5* in tomato has been reported to bind to DRE elements (ACCGAC) within the promoters of *SlATG8d* and *SlATG18h*, inducing their expression and enhancing autophagic activity under drought conditions, which contributes to increased drought tolerance [44]. In *Chrysanthemum*, the promoter regions of *CmATG* family genes are enriched with W-boxes, TGACG motifs, and MYB binding sites, providing potential interaction sites for WRKY, TGA, and MYB transcription factors. The presence of these cis-elements suggests that *CmATG* genes may exhibit tissue-specific and stress-responsive expression patterns, which could be critical for modulating autophagy under different environmental conditions. This highlights a promising direction for further research into the upstream regulatory networks of *CmATG* genes, providing insights into their roles in stress adaptation and potential applications in molecular breeding for stress resilience.

Research in *A. thaliana* has shown that *AtATG* genes are not tissue-specific [45]. However, the four genes *AtATG8a*, *AtATG8c*, *AtATG8f*, and *AtATG8i* are widely expressed across various developmental stages and tissues [46]. Similarly, expression analysis in *N. tabacum* revealed that *NtATG* genes are present in roots, stems, leaves, pollen, anthers, and seeds, with the highest expression levels observed in pollen and anthers, particularly within the *NtATG8* subfamily [33]. Consistent with these findings, most *CmATG* genes were found to exhibit stable expression across different tissues. However, *CmATG8b*, *CmATG8n*, *CmATG8d*, and *CmATG8i* showed notably high transcription levels and tissue-specific expression, similar to the patterns observed in *Arabidopsis*, *Nicotiana*, and *Solanum*. The widespread expression of *ATG8* subfamily genes in plant tissues suggests their essential role in autophagy throughout plant development and stress responses [34].

The response of *CmATG8* genes to heat stress was assessed by analyzing their expression following exposure to 40 °C for 48 h. Members of the *CmATG8* subfamily were markedly induced under heat stress, with expression levels generally increasing over time and reaching their maximum at 48 h. Notably, *CmATG8o* exhibited its highest expression at 72 h. Similar heat-responsive expression patterns have been observed in *Capsicum annuum*, where most *CaATG* genes peaked at 1 h of heat treatment, *CaATG8c* and *CaATG18b* peaked at 3 h and 6 h, respectively, and nearly all genes returned to baseline levels after 12 h [23]. In *Gossypium hirsutum*, *GhATG8fA* showed peak expression at 1 h of heat stress, while *GhATG8eD* and *GhATG8cD* reached their highest expression at 24 h [7]. The expression dynamics of *CmATG8* genes under heat stress closely resembled those of *CaATG8* and *GhATG8*, suggesting that *CmATG8* genes play a crucial role in chrysanthemum’s heat stress response. *CmATG8o* was identified as a strong candidate gene for future studies on the autophagy-related mechanisms in *Chrysanthemum*.

This study provides a comprehensive genome-wide characterization of the *ATG* gene family in *Chrysanthemum*, including bioinformatic analyses, expression profiling under heat stress, and preliminary functional validation of the *CmATG8* subfamily. These findings lay a foundation for future studies on the *CmATG* gene family and offer valuable genetic resources for breeding heat tolerant chrysanthemum cultivars. Furthermore, the polyploid *CmATG* gene family analysis serves as a reference for *ATG* gene studies in other polyploid plant species.

## 4. Materials and Methods

### 4.1. Identification of ATG Gene Family Members

The genomic coding sequence (CDS) and protein sequence data, along with annotation files for *C. morifolium*, *C. lavandulifolium*, and *C. nankingense*, were retrieved from the *Chrysanthemum* Genome Database (http://210.22.121.250:8880/asteraceae/download/downloadPage) (accessed on 4 August 2024). Autophagy-related genes in these species were identified by using the reported *A. thaliana* ATG protein sequences as references, followed by BLASTp searches across the three genomes performed in TBtools. The search parameters were set with an E-value threshold of <1 × 10^−5^ and a sequence similarity of at least 60%.

After removing redundant sequences, the identified candidate *ATGs* (*CmATGs*, *ClATGs*, and *CnATGs*) were further analyzed for conserved domains using multiple databases, including PFAM (https://pfam.xfam.org/search/) (accessed on 10 August 2024), NCBI-CDD Search (https://plants.ensembl.org/) (accessed on 10 August 2024), and SMART (http://smart.embl-heidelberg.de/) (accessed on 10 August 2024). Only genes containing conserved *ATG* domains were retained as final *ATG* gene family members. Additionally, key physicochemical properties of the identified *ATG* proteins, such as molecular weight and isoelectric point, were predicted using the ExPASy tool (https://web.expasy.org/) (accessed on 12 August 2024).

### 4.2. Phylogenetic Tree Construction of ATG Gene Family

Multiple sequence alignment of the *ATG* protein sequences from *C. morifolium*, *C. nankingense*, *C. lavandulifolium*, and Arabidopsis was performed using the Clustal W method in MEGA 11.0. The Neighbor-Joining (NJ) method was used to construct the phylogenetic tree, with a bootstrap value of 1000 replicates.

### 4.3. Gene Structure, Conserved Motif, and Domain Analysis of ATG Gene Family

Gene structure analysis and visualization were performed using GSDS 2.0 (https://gsds.gao-lab.org/) (accessed on 22 August 2024) based on the coding sequences and full-length sequences of the *ATG* gene family members. MEME (https://meme-suite.org/meme/tools/meme) (accessed on 25 August 2024) was employed to identify conserved motifs in *CmATG* proteins, with the motif number set to 20. TBtools was used for visualizing protein domains in combination with the motif data.

### 4.4. Chromosomal Localization and Synergy Analysis of ATG Gene Family Members

The GFF3 annotation files for each genome were imported into TBtools. The IDs of the identified ATG genes were input, and a map depicting the distribution and localization on the chromosomes of *Chrysanthemum* was constructed. Synteny relationships of ATG genes between *C. morifolium*, *C. nankingense*, and *C. lavandulifolium* was conducted using MCScanX in TBtools. The results were visualized using TBtools’ Advanced Circos and Multiple Synteny Plot.

### 4.5. Analysis of Cis-Regulatory Elements in the Promoters of the Chrysanthemum ATG Gene Family

The 2000 bp upstream of the start codon of each *ATG* gene family member was extracted from the respective genomes and submitted to PlantCARE (https://bioinformatics.psb.ugent.be/webtools/plantcare/html/) (accessed on 27 August 2024) for prediction of cis-acting elements. The results were compiled and visualized using TBtools.

### 4.6. Tissue-Specific Expression of the Chrysanthemum ATG Gene Family

Expression data for all identified *CmATG* genes in different tissues were retrieved from the *Chrysanthemum* transcriptome database (http://210.22.121.250:8880/asteraceae/rna/searchPage) (accessed on 30 August 2024). The expression levels of these genes in various tissues were imported into TBtools to generate a heatmap.

### 4.7. Expression Analysis of CmATG8 Subfamily in Response to Heat Stress

*Chrysanthemum* cultivar ‘Jinba’ tissue culture plantlets were used as experimental materials. After transplanting into pots, the plantlets were grown in a controlled growth chamber under the following conditions: 25 °C/22 °C (day/night), 5000 lux light intensity, and a 14 h/10 h light/dark photoperiod. When the plants developed 8–10 fully expanded leaves, they were subjected to heat stress at 40 °C. Leaf samples were collected at 0, 1, 3, 6, 12, 24, 48, and 72 h after treatment. Total RNA was extracted from the leaves and reverse transcribed into cDNA. Gene specific primers (Supplementary Table S4) were designed for qRT-PCR analysis. *CmEF1α* was used as the internal reference gene. Prior to conducting inferential tests, data normality was assessed using the Shapiro–Wilk test, and homogeneity of variances was evaluated with Levene’s test in IBM SPSS 22.0. Relative gene expression levels were calculated using the 2^−ΔΔCt^ method. Data meeting the assumptions of normality and equal variances were analyzed by one-way ANOVA followed by Duncan’s multiple range test at a significance level of *p* < 0.05 using DPS 9.01. Bar graphs were generated with Origin 2022. Results are presented as mean ± SD.

## 5. Conclusions

A total of 130, 51, and 49 *ATG* genes were identified in *C. morifolium*, *C. nankingense*, and *C. lavandulifolium*, with uneven chromosomal distribution. Collinearity analysis indicated a closer evolutionary relationship between *C. morifolium* and *C. lavandulifolium*, while phylogenetic and conserved motif analyses revealed subfamily-specific conservation and structural diversity. Transcriptomic profiling showed broad expression of *CmATG* genes, with floral buds exhibiting the highest levels and strong heat-induced upregulation in the *ATG8* subfamily, particularly *CmATG8o*.

The identification of heat-responsive *CmATG* genes, particularly within the *ATG8* subfamily, provides promising genetic targets for enhancing heat tolerance in chrysanthemum through molecular breeding or genome-editing. However, the functional roles of ATG proteins in autophagosome biogenesis, protein–protein interactions, and metabolic reprogramming under stress remain unclear. Future studies should prioritize functional validation of key genes and elucidation of the associated signaling pathways and regulatory networks.

## Data Availability

The datasets generated or analyzed during the current study are available from the corresponding author on reasonable request.

## Supplementary Materials

The following supporting information can be downloaded at: https://www.mdpi.com/article/10.3390/ijms26178642/s1.
