# Supplementary material for: Genome-Wide Identification of Autophagy-Related Gene Family and Gene Expression Analysis of the CmATG8 Under Heat Stress in Chrysanthemum
Source: Int J Mol Sci. 2025 Sep 5;26(17):8642. doi: 10.3390/ijms26178642 (PMC12428883; doi:10.3390/ijms26178642)
Supplement: Supplementary file 1 [file ijms-26-08642-s001.zip › Figure S1-S8.pdf]

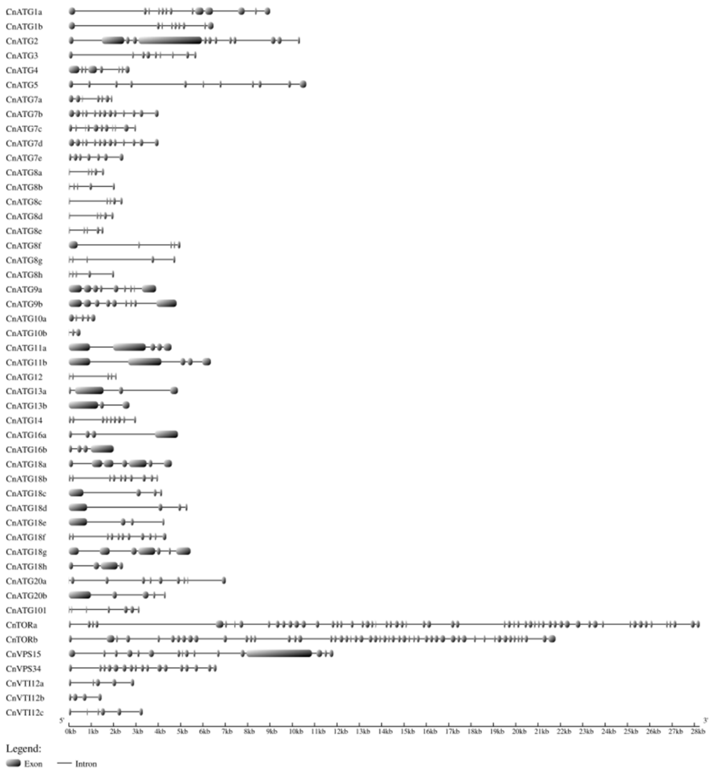

Figure S1. Gene structure of *CnATGs*. The exon-intron structures, with exons in black rectangles and introns in thin black lines.

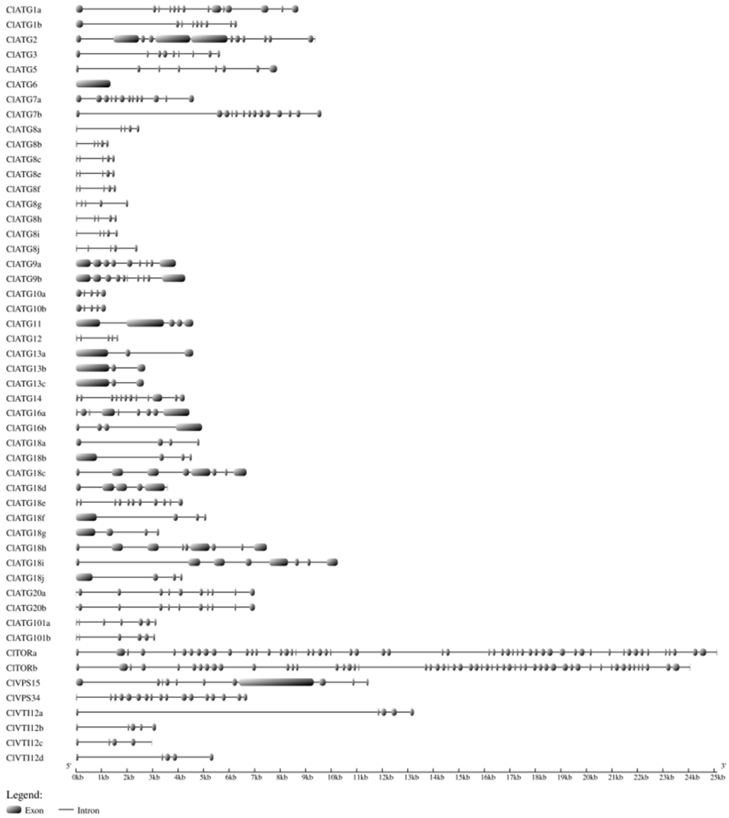

Figure S2. Gene structure of *ClATGs.*The exon-intron structures, with exons in black rectangles

and introns in thin black lines.

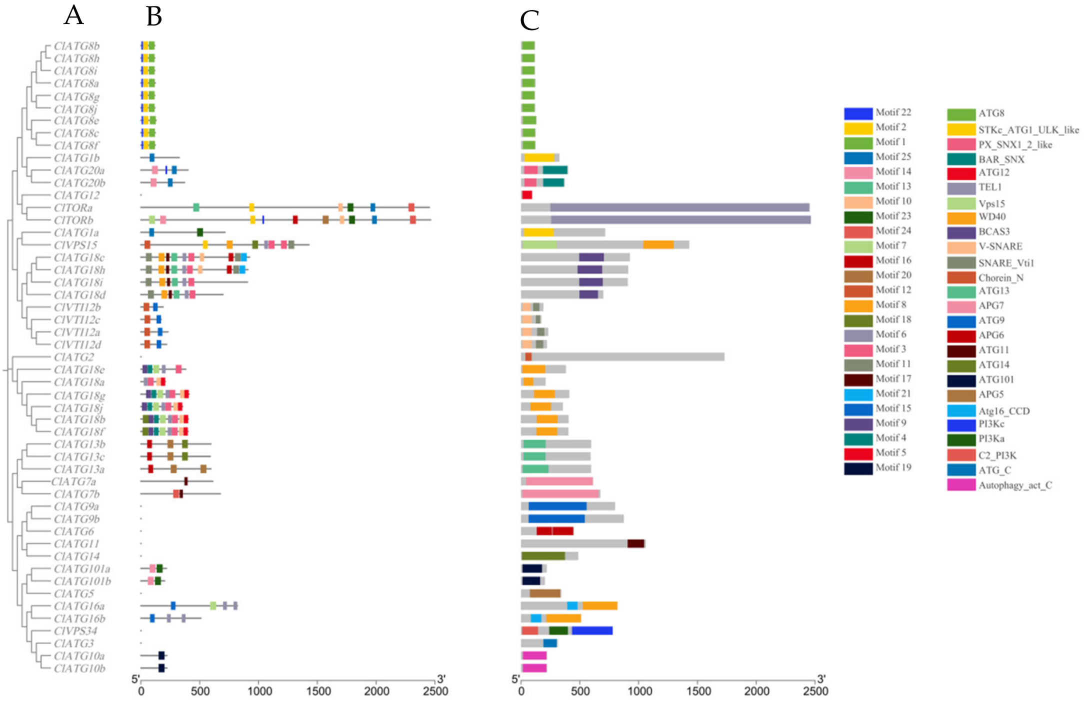

Figure S3. Analysis of ClATG protein domain and conserved motif. (A) Phylogenetic tree of the *ClATG* gene family. (B) Motifs of ClATG proteins. (C) Functional domains distribution of ClATGs.

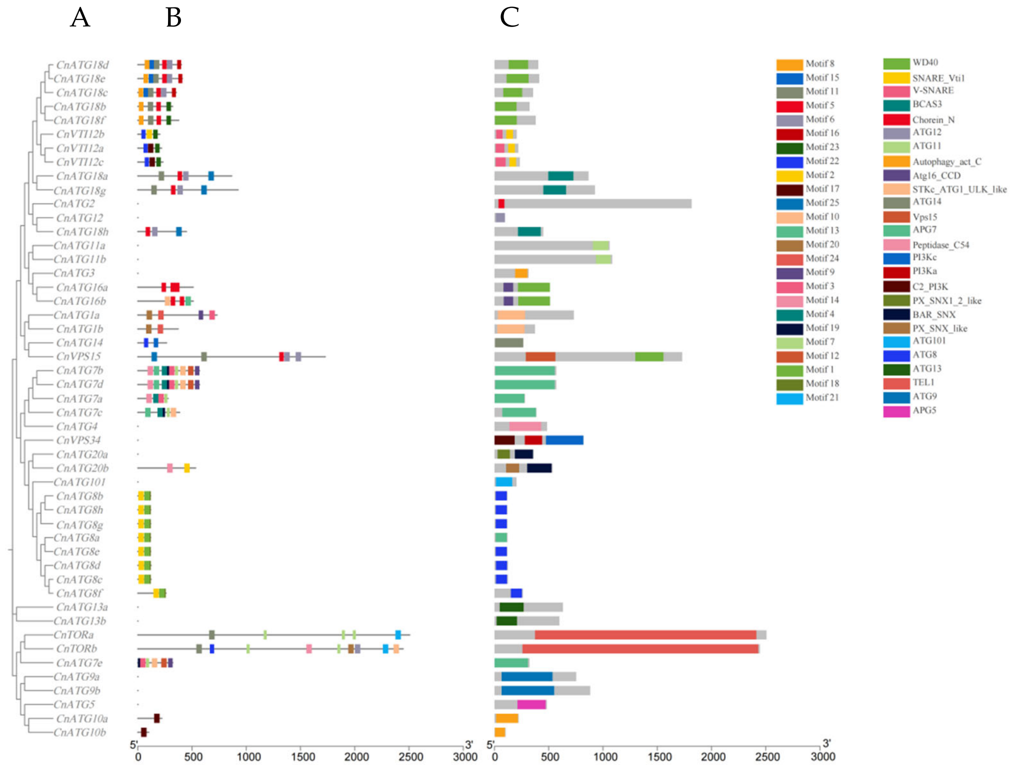

Figure S4. Analysis of CnATG protein domain and conserved motif. (A) Phylogenetic tree of the

CnATG gene family. (B) Motifs of CnATG proteins. (C) Functional domains distribution of CnATGs.

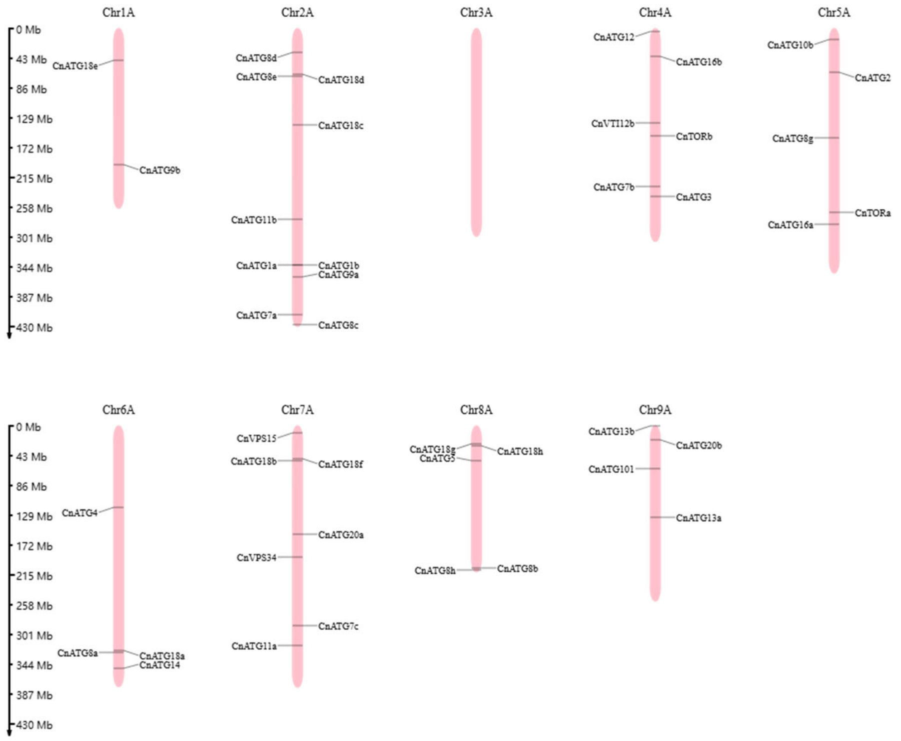

Figure S5. Chromosomal distribution of *CnATGs* in *C. nankingense*. A total of 51 *CnATGs* were mapped onto the 9 chromosomes. The gene names are labeled in black. The scale on the left represents chromosome length in megabases (Mb). The gene positions were determined based on the reference genome annotation.

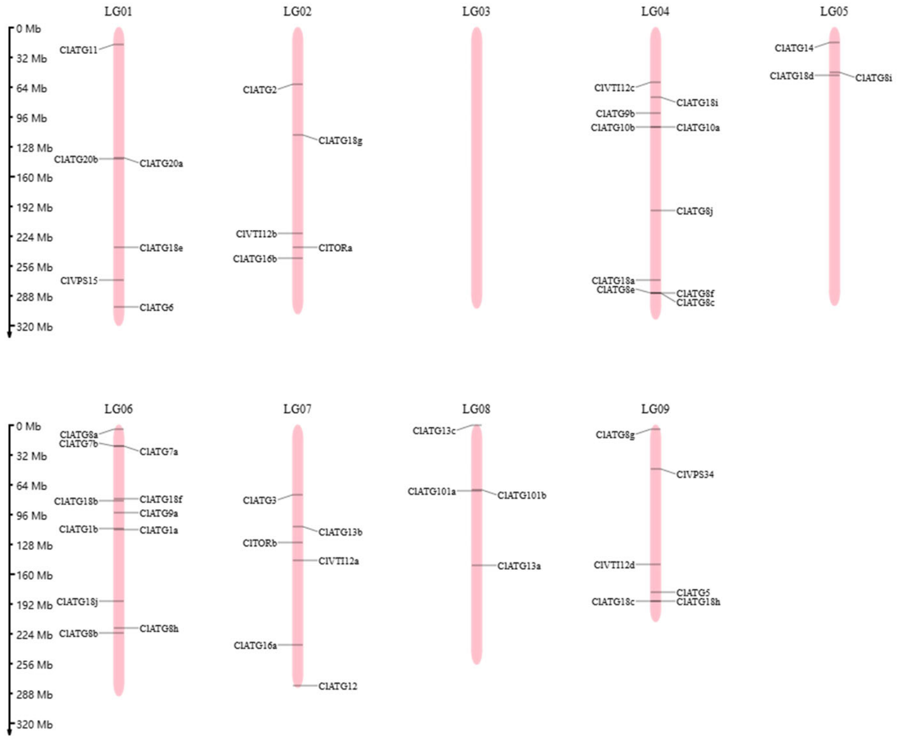

Figure S6. Chromosomal distribution of *ClATGs* in *C. lavandulifolium*. A total of 49 *ClATGs* were mapped onto the 9 chromosomes. The gene names are labeled in black. The scale on the left represents chromosome length in megabases (Mb). The gene positions were determined based on the reference genome annotation.

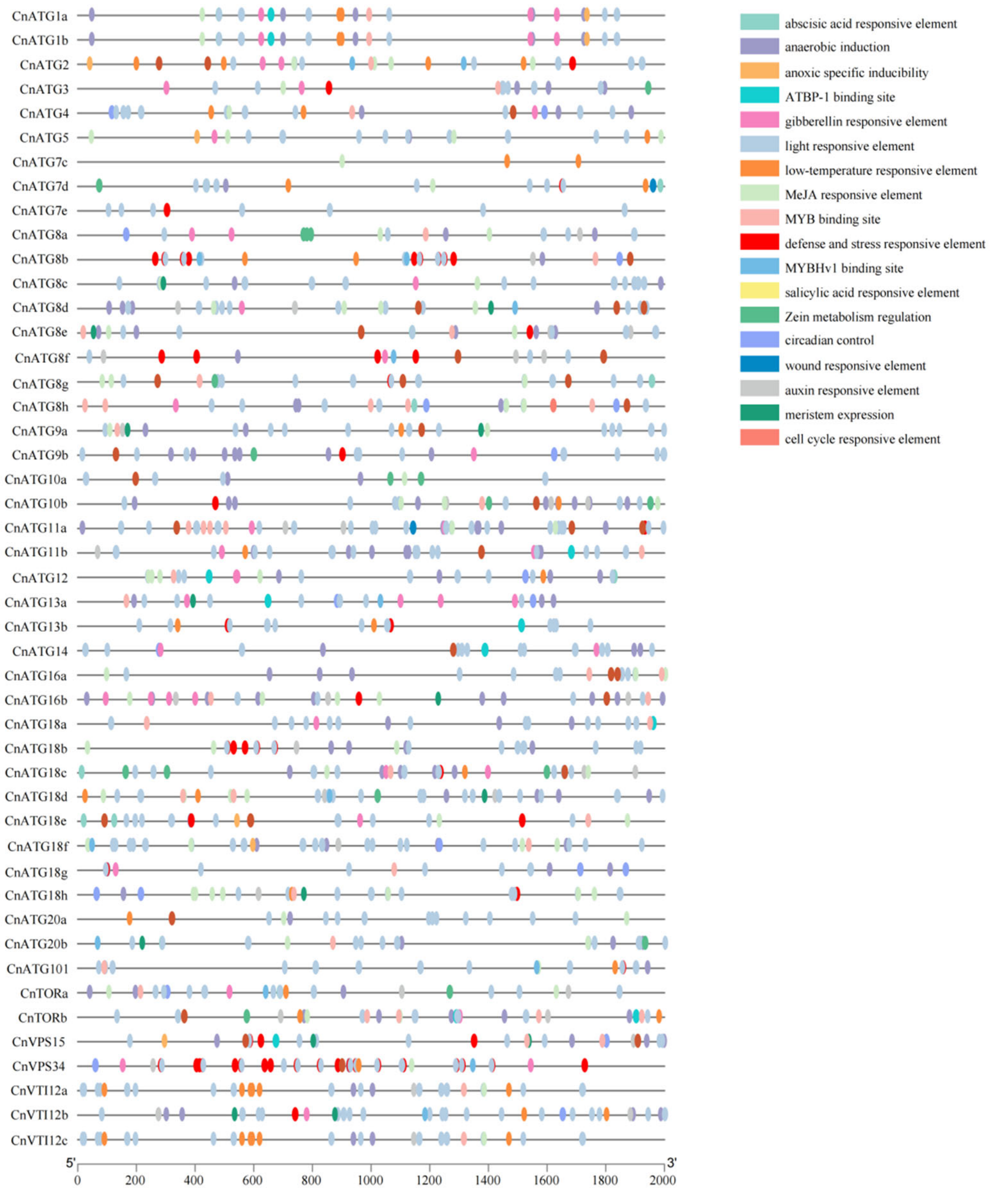

Figure S7. Analysis of cis-acting regulatory elements in the promoter regions of *CnATG* genes. The distribution and abundance of different cis-regulatory element types are shown for each gene. Promoter sequences comprising 2000 bp upstream of the predicted translation start site (ATG) were retrieved and analyzed using PlantCARE.

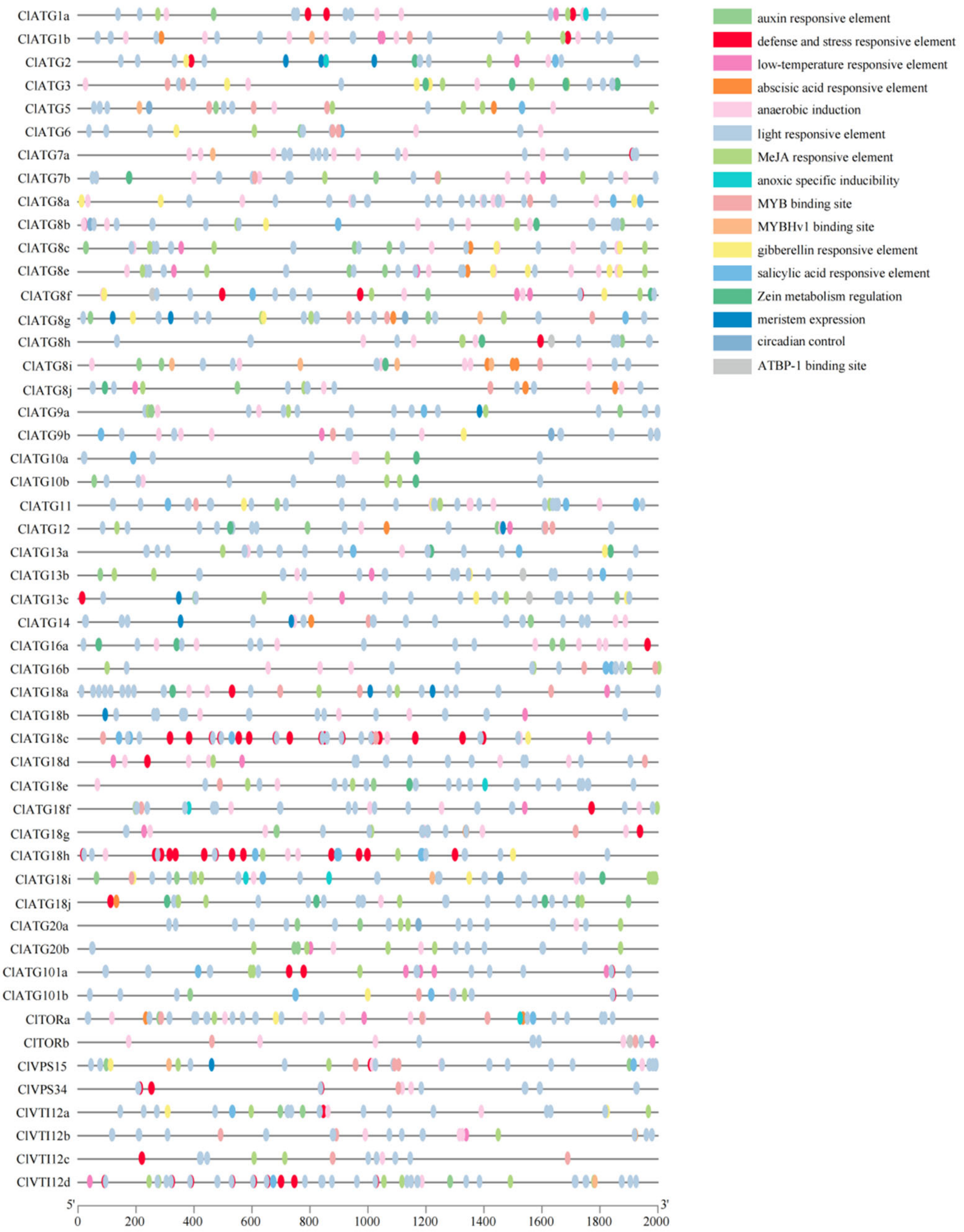

Figure S8. Analysis of cis-acting regulatory elements in the promoter regions of *ClATG* genes. The distribution and abundance of different cis-regulatory element types are shown for each gene. Promoter sequences comprising 2000 bp upstream of the predicted translation start site (ATG) were retrieved and analyzed using PlantCARE.
