# Supplementary material for: Genome-Wide Identification of Autophagy-Related Gene Family and Gene Expression Analysis of the CmATG8 Under Heat Stress in Chrysanthemum
Source: Int J Mol Sci. 2025 Sep 5;26(17):8642. doi: 10.3390/ijms26178642 (PMC12428883; doi:10.3390/ijms26178642)
Supplement: Supplementary file 1 [file ijms-26-08642-s001.zip › Table S1.pdf]

Table S1. Bioinformatics analysis of *CmATG* proteins

Gene name	Gene ID	Number of Amino Acid	Molecular Weight	Theoretical pI	Instability Index	Aliphatic Index	Grand Average of Hydropathicity
CmATG1a	evm.TU.scaffold_790.2	732	81434.2Da	6.68	59.52	83.01	-0.458
CmATG1b	evm.TU.scaffold_1587.128	376	42884.53Da	9.32	60.82	88.64	-0.434
CmATG1c	evm.TU.scaffold_1587.134	728	80834.57Da	6.66	58.94	83.34	-0.434
CmATG2a	evm.TU.scaffold_10264.315	1831	201659.82Da	5.83	42.1	88.3	-0.23
CmATG2b	evm.TU.scaffold_11705.63	1831	201539.62Da	5.8	41.86	88.09	-0.231
CmATG3a	evm.TU.scaffold_asm3_new.563	314	35631.77Da	4.58	46.71	81.31	-0.54
CmATG3b	evm.TU.scaffold_11087.4	314	35631.77Da	4.58	46.71	81.31	-0.54
CmATG3c	evm.TU.scaffold_1252.152	314	35585.74Da	4.58	47.37	81.62	-0.519
CmATG4a	evm.TU.scaffold_1542.78	479	53127.47Da	5.04	52.84	74.72	-0.362
CmATG4b	evm.TU.scaffold_10104.51	480	53177.52Da	5.19	54.09	75.17	-0.349
CmATG4c	evm.TU.scaffold_11315.19	479	53217.58Da	5.13	52.48	74.3	-0.359
CmATG5a	evm.TU.scaffold_407.81	343	38727.47Da	5.35	39.38	95.51	-0.199
CmATG5b	evm.TU.scaffold_1764.130	432	48621.8Da	5.31	43.97	94.79	-0.222
CmATG5c	evm.TU.scaffold_7753.59	343	38757.56Da	5.26	38.65	96.09	-0.171
CmATG6a	evm.TU.scaffold_1046.476	522	59398.96Da	6.45	40.96	68.77	-0.655
CmATG6b	evm.TU.scaffold_1473.51	457	52596.64Da	5.71	44.99	72.56	-0.593
CmATG6c	evm.TU.scaffold_6398.18	522	59427.02Da	6.63	39.99	68.77	-0.657
CmATG6d	evm.TU.scaffold_asm20_new.1170	522	59427.02Da	6.63	39.99	68.77	-0.657
CmATG7a	evm.TU.scaffold_490.247	147	15927.8Da	8.32	36.88	102.18	0.129
CmATG7b	evm.TU.scaffold_490.248	202	21898.48Da	4.79	38.86	89.31	-0.194

CmATG7c	evm.TU.scaffold_934.102	147	15872.72Da	8.32	37.46	100.88	0.131
CmATG7d	evm.TU.scaffold_1089.15	270	29059.69Da	5.62	46.42	106.59	0.185
CmATG7e	evm.TU.scaffold_1089.27	711	78238.88Da	5.14	49.42	89.73	-0.135
CmATG7f	evm.TU.scaffold_1149.112	215	23384.04Da	8.67	47.06	99.35	0.026
CmATG7g	evm.TU.scaffold_1442.31	262	28484Da	7.53	48.44	95.34	0.161
CmATG7h	evm.TU.scaffold_1827.180	369	40850.79Da	6.09	42.3	94.12	-0.093
CmATG7i	evm.TU.scaffold_6921.43	744	82079.4Da	5.73	48.87	88.24	-0.197
CmATG7j	evm.TU.scaffold_8801.49	710	78052.71Da	5.19	49.72	89.72	-0.138
CmATG8a	evm.TU.scaffold_183.43	119	13691.8Da	9.27	36.03	87.65	-0.463
CmATG8b	evm.TU.scaffold_309.134	120	13820.91Da	9.02	30.53	83.67	-0.446
CmATG8c	evm.TU.scaffold_320.107	180	20770.07Da	8.39	43.36	86.06	-0.407
CmATG8d	evm.TU.scaffold_490.117	119	13727.99Da	8.78	37.46	91.01	-0.316
CmATG8e	evm.TU.scaffold_647.81	119	13621.62Da	8.75	43.37	85.29	-0.497
CmATG8f	evm.TU.scaffold_762.156	120	13730.79Da	9.05	31.23	82.83	-0.444
CmATG8g	evm.TU.scaffold_875.111	119	13727.99Da	8.78	37.46	91.01	-0.316
CmATG8h	evm.TU.scaffold_891.230	119	13721.79Da	9.07	38.91	85.21	-0.528
CmATG8i	evm.TU.scaffold_891.272	119	13721.79Da	9.07	38.91	85.21	-0.528
CmATG8j	evm.TU.scaffold_985.72	119	13713.97Da	8.78	41.15	91.01	-0.317
CmATG8k	evm.TU.scaffold_1298.127	123	14197.47Da	7.77	45.28	83.17	-0.388
CmATG8l	evm.TU.scaffold_1403.172	123	14197.47Da	7.77	45.28	83.17	-0.388
CmATG8m	evm.TU.scaffold_1644.213	119	13650.66Da	7.84	38.68	86.89	-0.477
CmATG8n	evm.TU.scaffold_2891.105	119	13727.99Da	8.78	37.46	91.01	-0.316
CmATG8o	evm.TU.scaffold_4753.8	119	13713.97Da	8.78	41.15	91.01	-0.317
CmATG8p	evm.TU.scaffold_8056.31	182	20674.29Da	8.46	38.19	98.08	0.049

CmATG8q	evm.TU.scaffold_11771.381	119	13650.66Da	7.84	37.97	86.89	-0.477
CmATG9a	evm.TU.scaffold_62.98	800	92107.29Da	6.5	45.23	86.12	-0.242
CmATG9b	evm.TU.scaffold_582.74	799	92082.51Da	7.63	50.45	86.23	-0.223
CmATG9c	evm.TU.scaffold_858.465	799	92317.74Da	7.13	48.04	85.86	-0.229
CmATG9d	evm.TU.scaffold_1015.77	800	92165.3Da	6.38	45.34	85.51	-0.237
CmATG9e	evm.TU.scaffold_1709.120	889	101786.77Da	5.82	43.09	78.58	-0.267
CmATG9f	evm.TU.scaffold_3601.196	889	101807.75Da	5.82	44.33	78.14	-0.271
CmATG10a	evm.TU.scaffold_289.5	264	30349.11Da	5.92	41.05	74.92	-0.496
CmATG10b	evm.TU.scaffold_460.279	220	25244.07Da	5.35	42.11	66.91	-0.608
CmATG10c	evm.TU.scaffold_1234.64	229	26288.39Da	5.37	32.6	71.97	-0.557
CmATG10d	evm.TU.scaffold_2603.1	220	25244.07Da	5.35	42.11	66.91	-0.608
CmATG10e	evm.TU.scaffold_6206.94	86	9847.33Da	7.79	42.47	73.72	-0.265
CmATG11a	evm.TU.scaffold_205.127	1061	119545.24Da	5.55	43.01	83.36	-0.486
CmATG11b	evm.TU.scaffold_391.123	1061	119609.3Da	5.5	42.88	83	-0.487
CmATG11c	evm.TU.scaffold_1151.112	1084	122487.89Da	5.49	42.89	81.34	-0.585
CmATG11d	evm.TU.scaffold_3674.167	1084	122831.31Da	5.45	42.71	81.79	-0.595
CmATG11e	evm.TU.scaffold_6083.293	1061	119512.14Da	5.61	42.85	83.45	-0.49
CmATG11f	evm.TU.scaffold_asm13_new.2117	1084	122831.31Da	5.45	42.71	81.79	-0.595
CmATG12a	evm.TU.scaffold_339.127	97	10761.32Da	9.4	49.98	84.43	-0.133
CmATG12b	evm.TU.scaffold_911.129	95	10567.09Da	9.4	45.87	86.21	-0.095
CmATG12c	evm.TU.scaffold_11961.23	92	10277.84Da	9.63	43.78	89.02	-0.05
CmATG13e	evm.TU.scaffold_608.28	590	65487.95Da	9.14	76.15	65.44	-0.633
CmATG13c	evm.TU.scaffold_844.15	597	65973.32Da	9.12	75.74	66.62	-0.579

CmATG13a	evm.TU.scaffold_5394.90	591	65629.21Da	9.2	76.48	65.33	-0.628
CmATG13b	evm.TU.scaffold_9671.148	597	65947.24Da	9.12	76.16	65.96	-0.587
CmATG13d	evm.TU.scaffold_10341.45	597	66022.39Da	9.12	75.55	66.62	-0.575
CmATG14a	evm.TU.scaffold_1730.14	487	54148.36Da	9.1	41.38	78.34	-0.479
CmATG14b	evm.TU.scaffold_2548.350	487	54219.44Da	9.15	41.69	77.74	-0.491
CmATG14c	evm.TU.scaffold_8521.30	488	54291.43Da	9.05	43.01	76.58	-0.487
CmATG16a	evm.TU.scaffold_1115.128	511	56378.37Da	6.17	41.44	82.49	-0.408
CmATG16b	evm.TU.scaffold_1371.37	515	56770.06Da	6.27	33.65	88.85	-0.32
CmATG16c	evm.TU.scaffold_1620.162	511	56321.26Da	6.17	41.58	81.92	-0.413
CmATG16e	evm.TU.scaffold_9643.36	511	56426.42Da	6.24	42.45	80.96	-0.429
CmATG16f	evm.TU.scaffold_9671.23	919	103532.49Da	7.56	40.05	84.36	-0.441
CmATG16g	evm.TU.scaffold_10124.247	514	56687.87Da	6.23	35.52	85.99	-0.355
CmATG18a	evm.TU.scaffold_203.53	865	94537.85Da	5.97	45.79	78.5	-0.426
CmATG18b	evm.TU.scaffold_296.56	356	39666.38Da	6.7	33.26	94.38	-0.002
CmATG18c	evm.TU.scaffold_459.49	924	101158.43Da	5.54	53.21	74.52	-0.392
CmATG18d	evm.TU.scaffold_553.209	356	39671.35Da	6.5	34.95	94.94	-0.002
CmATG18e	evm.TU.scaffold_679.124	406	44968.01Da	8.42	33.33	78.92	-0.227
CmATG18f	evm.TU.scaffold_838.168	411	45818.04Da	7.61	44.58	83.45	-0.168
CmATG18g	evm.TU.scaffold_903.203	381	41305.96Da	7.16	36.33	98.06	0.167
CmATG18h	evm.TU.scaffold_1074.51_evm.TU.scaffold_1074.50	907	99107.3Da	6.05	45.74	80.32	-0.318
CmATG18i	evm.TU.scaffold_1101.21	360	40031.72Da	6.5	32.08	93.89	-0.008
CmATG18j	evm.TU.scaffold_1139.487	429	47704.19Da	7.61	46.72	84.5	-0.146
CmATG18k	evm.TU.scaffold_1201.94	380	41199.79Da	7.13	35.32	97.55	0.149

CmATG18l	evm.TU.scaffold_1430.279	907	99106.25Da	5.97	45.41	79.68	-0.331
CmATG18m	evm.TU.scaffold_1465.32	924	101172.56Da	5.58	53.51	75.04	-0.377
CmATG18n	evm.TU.scaffold_1573.433	405	45015.62Da	6.91	36.44	72.15	-0.338
CmATG18o	evm.TU.scaffold_1573.833	405	44956.55Da	6.91	40.39	72.15	-0.334
CmATG18p	evm.TU.scaffold_1578.94	380	41250.84Da	6.82	35.66	97.55	0.141
CmATG18q	evm.TU.scaffold_1583.64	410	45179.28Da	8.39	33.74	78.88	-0.188
CmATG18r	evm.TU.scaffold_1654.292	401	44602.18Da	6.91	38.97	72.87	-0.325
CmATG18s	evm.TU.scaffold_1668.85	869	94790.12Da	5.97	46.82	78.7	-0.421
CmATG18t	evm.TU.scaffold_1797.94	412	45545.84Da	8.76	32.48	80.85	-0.208
CmATG18u	evm.TU.scaffold_5290.12	865	94546.81Da	5.97	46.55	77.71	-0.431
CmATG18v	evm.TU.scaffold_5740.24	924	101231.54Da	5.54	53.11	74.52	-0.392
CmATG18w	evm.TU.scaffold_8085.271	411	45852.05Da	7.61	45.52	83.45	-0.167
CmATG18x	evm.TU.scaffold_asm13_new.12	360	40031.72Da	6.5	32.08	93.89	-0.008
CmATG20a	evm.TU.scaffold_737.46	406	46833.42Da	7.15	43.81	81.01	-0.602
CmATG20b	evm.TU.scaffold_1398.152	406	46821.43Da	6.79	42.18	81.01	-0.591
CmATG20c	evm.TU.scaffold_1569.196	535	59659.15Da	5.71	39.13	80.17	-0.531
CmATG20d	evm.TU.scaffold_1659.323	536	59846.31Da	5.77	41.33	80.73	-0.55
CmATG20e	evm.TU.scaffold_1799.28	535	59646.15Da	5.71	39.2	80.34	-0.526
CmATG20f	evm.TU.scaffold_7966.40	534	61323.2Da	8.56	50.81	88.76	-0.178
CmATG101a	evm.TU.scaffold_2080.103	218	25469.17Da	6	31.31	83.99	-0.421
CmATG101b	evm.TU.scaffold_6764.41	218	25469.17Da	6	31.31	83.99	-0.421
CmTORa	evm.TU.scaffold_764.556	2317	261211.51Da	6.52	44.19	99.82	-0.118
CmTORb	evm.TU.scaffold_1080.68	2317	261244.64Da	6.54	44.46	99.91	-0.115

CmTORc	evm.TU.scaffold_1451.358	2317	261310.64Da	6.55	44.22	99.82	-0.12
CmTORd	evm.TU.scaffold_4782.119	2464	276398.94Da	6.56	42.53	99.94	-0.113
CmVPS15a	evm.TU.scaffold_972.181	1456	162839.1Da	6.3	52.87	86.9	-0.246
CmVPS15b	evm.TU.scaffold_1267.34	1518	170413.93Da	6.58	52.74	86.61	-0.258
CmVPS15c	evm.TU.scaffold_1267.146	1447	162206.65Da	6.53	53.91	87.71	-0.232
CmVPS34a	evm.TU.scaffold_748.14	823	94189.3Da	6.28	43.67	92.52	-0.313
CmVPS34b	evm.TU.scaffold_949.32	823	94162.28Da	6.28	43.77	92.52	-0.309
CmVPS34c	evm.TU.scaffold_6090.79	823	94189.3Da	6.28	43.67	92.52	-0.313
CmVTI12a	evm.TU.scaffold_19.13	220	24915.67Da	8.77	53.7	94.05	-0.405
CmVTI12b	evm.TU.scaffold_397.54	221	24967.8Da	9.51	55.54	100.18	-0.416
CmVTI12c	evm.TU.scaffold_1097.261	220	24604.34Da	9.47	47.09	102.45	-0.4
CmVTI12d	evm.TU.scaffold_1116.207	220	24604.34Da	9.47	47.09	102.45	-0.4
CmVTI12e	evm.TU.scaffold_1166.79	220	24915.67Da	8.77	53.7	94.05	-0.405
CmVTI12f	evm.TU.scaffold_1300.52	221	24994.83Da	9.51	54.67	100.18	-0.425
CmVTI12g	evm.TU.scaffold_1366.118	220	24915.71Da	8.97	52.71	94.05	-0.403
CmVTI12h	evm.TU.scaffold_1425.69	220	24604.34Da	9.47	47.09	102.45	-0.4
CmVTI12i	evm.TU.scaffold_8157.89	221	24967.8Da	9.51	55.54	100.18	-0.416
