# Supplementary material for: Genome-Wide Identification of Autophagy-Related Gene Family and Gene Expression Analysis of the CmATG8 Under Heat Stress in Chrysanthemum
Source: Int J Mol Sci. 2025 Sep 5;26(17):8642. doi: 10.3390/ijms26178642 (PMC12428883; doi:10.3390/ijms26178642)
Supplement: Supplementary file 1 [file ijms-26-08642-s001.zip › Table S2.pdf]

Table S2. Bioinformatics analysis of *CnATG* proteins

Gene name	Gene ID	Number of Amino Acid	Molecular Weight	Theoretical pI	Instability Index	Aliphatic Index	Grand Average of Hydropathicity
CnATG1a	Cn0353580	731	81346.14Da	6.79	59.68	83.26	-0.455
CnATG1b	Cn0353600	373	42578.16Da	9.24	60.87	88.31	-0.406
CnATG2	Cn0048510	1816	199622.49Da	5.82	41.25	88.44	-0.211
CnATG3	Cn0746620	314	35629.8Da	4.58	46.23	81.62	-0.526
CnATG4	Cn0689760	484	53686.11Da	5.09	51.85	74.55	-0.366
CnATG5	Cn1031330	481	54327.7Da	5.74	42.29	98.09	-0.155
CnATG7a	Cn0223150	280	30839.72Da	8.76	42.99	85.71	-0.099
CnATG7b	Cn0590830	570	62721.55Da	5.48	43.37	91.89	-0.11
CnATG7c	Cn0703990	386	43060.83Da	8.78	38.59	98.01	-0.072
CnATG7d	Cn0923140	570	62687.53Da	5.48	43.23	92.58	-0.108
CnATG7e	Cn1063460	323	34977.06Da	5.27	39.01	97.18	-0.012
CnATG8a	Cn0220990	119	13713.97Da	8.78	41.15	91.01	-0.317
CnATG8b	Cn0520950	119	13650.66Da	7.84	37.97	86.89	-0.477
CnATG8c	Cn0537920	123	14197.47Da	7.77	45.28	83.17	-0.388
CnATG8d	Cn0777960	123	14139.44Da	8.61	45.28	83.98	-0.345
CnATG8e	Cn0813650	119	13727.99Da	8.78	37.46	91.01	-0.316
CnATG8f	Cn0834370	261	30638.63Da	4.97	56.11	58.28	-1.079
CnATG8g	Cn0948990	119	13721.79Da	9.07	38.91	85.21	-0.528
CnATG8h	Cn1182910	119	13636.63Da	7.84	37.97	86.05	-0.48
CnATG9a	Cn0796450	753	87239.75Da	6.3	44.52	85.92	-0.256
CnATG9b	Cn0827260	882	100912.85Da	5.91	43.92	78.88	-0.26
CnATG10a	Cn0076430	222	25296.14Da	5.13	35.02	65.45	-0.559
CnATG10b	Cn0442210	101	11602.2Da	5.26	46.13	74.36	-0.399
CnATG11a	Cn0428870	1061	119606.28Da	5.52	42.72	83	-0.492
CnATG11b	Cn0477050	1084	122743.22Da	5.45	42.16	81.61	-0.594
CnATG12	Cn1149940	96	10664.2Da	9.4	48.39	85.31	-0.118
CnATG13a	Cn1559490	630	69289.73Da	8.54	78.26	65.9	-0.567
CnATG13b	Cn1576040	598	66461.93Da	9.21	75.7	64.73	-0.664
CnATG14	Cn0970070	266	30218.76Da	9.56	35.14	90.19	-0.468
CnATG16a	Cn0906000	511	56335.29Da	6.17	41.59	81.92	-0.412
CnATG16b	Cn0941180	512	56374.39Da	6.21	36.39	85.96	-0.368
CnATG18a	Cn0219970	866	94612.87Da	5.97	46.41	78.07	-0.433
CnATG18b	Cn0388510	322	34537.31Da	7.64	37.52	97.24	0.192
CnATG18c	Cn0597560	356	39666.38Da	6.7	33.26	94.38	-0.002
CnATG18d	Cn0799190	403	44773.34Da	6.7	38.75	72.51	-0.33
CnATG18e	Cn0878220	412	45615.86Da	8.36	31.14	79.42	-0.195
CnATG18f	Cn1168040	378	41004.57Da	7.16	35.62	97.54	0.138
CnATG18g	Cn1297610	925	101886.93Da	5.63	55.04	77.3	-0.325
CnATG18h	Cn1561920	449	49508.47Da	6.54	52.36	81.56	-0.31

CnATG20a	Cn0639830	362	41632.51Da	8.86	40.74	78.98	-0.568
CnATG20b	Cn1027330	534	59511.84Da	5.58	40.42	79.03	-0.557
CnATG101	Cn1109860	203	23634.12Da	6.14	30.78	85.86	-0.391
CnTORa	Cn0633620	2507	281609.62Da	6.28	44.48	99.03	-0.113
CnTORb	Cn0082180	2446	274326.31Da	6.48	42.33	99.68	-0.129
CnVPS15	Cn0104490	1728	194291.21Da	7.98	50.06	88.11	-0.279
CnVPS34	Cn0670760	821	93911.03Da	6.26	43.57	92.74	-0.302
CnVTI12a	Cn0510900	220	24869.59Da	8.77	51.7	94.05	-0.42
CnVTI12b	Cn1208750	204	23158.94Da	9.92	57.64	103.24	-0.396
CnVTI12c	Cn1493450	232	26440.36Da	8.72	51.92	93.36	-0.424
