# Supplementary material for: Genome-Wide Identification of Autophagy-Related Gene Family and Gene Expression Analysis of the CmATG8 Under Heat Stress in Chrysanthemum
Source: Int J Mol Sci. 2025 Sep 5;26(17):8642. doi: 10.3390/ijms26178642 (PMC12428883; doi:10.3390/ijms26178642)
Supplement: Supplementary file 1 [file ijms-26-08642-s001.zip › Table S3.pdf]

Table S3. Bioinformatics analysis of *ClATG* proteins

Gene name	Gene ID	Number of Amino Acid	Molecular Weight	Theoretical pI	Instability Index	Aliphatic Index	Grand Average of Hydropathicity
ClATG1a	EVM0035950	716	79828.53Da	6.66	59.46	83.65	-0.447
ClATG1b	EVM0049505	327	37562.75Da	9.88	55.41	91.53	-0.387
ClATG2	EVM0047396	1730	191203.26Da	5.75	42.51	89.33	-0.214
ClATG3	EVM0002051	314	35629.8Da	4.58	46.23	81.62	-0.526
ClATG5	EVM0027806	343	38725.45Da	5.27	38.92	95.8	-0.2
ClATG6	EVM0086776	451	51930.84Da	5.54	49.25	72.66	-0.598
ClATG7a	EVM0022985	612	68864Da	6.16	36.8	87.99	-0.055
ClATG7b	EVM0053606	676	74073.99Da	5.07	47.8	88.89	-0.149
ClATG8a	EVM0001212	123	14197.47Da	7.77	45.28	83.17	-0.388
ClATG8b	EVM0005970	119	13727.99Da	8.78	37.46	91.01	-0.316
ClATG8c	EVM0006722	123	14229.24Da	5.36	45.19	71.3	-0.408
ClATG8e	EVM0026135	131	15214.32Da	6.06	46.7	70.69	-0.488
ClATG8f	EVM0048828	123	14195.23Da	5.36	44.18	74.47	-0.394
ClATG8g	EVM0051325	119	13650.66Da	7.84	37.97	86.89	-0.477
ClATG8h	EVM0065425	119	13727.99Da	8.78	37.46	91.01	-0.316
ClATG8i	EVM0074848	119	13713.97Da	8.78	41.15	91.01	-0.317
ClATG8j	EVM0087271	120	13778.83Da	9.05	31.23	80.42	-0.456
ClATG9a	EVM0006534	800	92296.6Da	6.36	46.71	87.1	-0.226
ClATG9b	EVM0033467	874	99816.4Da	5.62	40.68	79.04	-0.248
ClATG10a	EVM0062439	222	25409.25Da	5.36	39.11	68.96	-0.616
ClATG10b	EVM0076708	222	25460.44Da	5.64	35.15	69.82	-0.573
ClATG11	EVM0023487	1061	119485.02Da	5.55	42.86	83.27	-0.492
ClATG12	EVM0057078	95	10567.09Da	9.4	46.77	86.21	-0.102
ClATG13a	EVM0033943	596	65959.54Da	9.26	76.78	68.52	-0.548
ClATG13b	EVM0052024	596	66073.56Da	9.2	76.66	64.78	-0.631
ClATG13c	EVM0083740	591	66108.98Da	9.38	77.79	65.47	-0.602
ClATG14	EVM0077983	487	54179.43Da	9.1	42.64	77.74	-0.481
ClATG16a	EVM0024544	821	92730.01Da	6.88	41.81	84.45	-0.448
ClATG16b	EVM0032303	511	56351.29Da	6.17	41.76	81.72	-0.417
ClATG18a	EVM0011853	209	23146.28Da	8.74	27.64	83.88	-0.182
ClATG18b	EVM0028280	405	44983.57Da	6.91	38.12	72.15	-0.34
ClATG18c	EVM0038734	925	101239.54Da	5.54	53.2	75.07	-0.389
ClATG18d	EVM0059907	699	75877.13Da	9.38	45.63	81.83	-0.298
ClATG18e	EVM0063155	382	41364.98Da	7.16	35.58	97.57	0.159
ClATG18f	EVM0065287	402	44649.24Da	6.91	36.33	72.69	-0.312
ClATG18g	EVM0073462	411	45862.09Da	7.61	45.05	83.45	-0.169
ClATG18h	EVM0079108	911	99885.94Da	5.58	53.39	74.41	-0.413
ClATG18i	EVM0082387	907	99142.43Da	6.08	44.68	80.53	-0.305
ClATG18j	EVM0082617	356	39657.37Da	6.67	34.43	94.38	-0.003

ClATG20a	EVM0028413	401	46259.82Da	7.73	42.52	81.52	-0.581
ClATG20b	EVM0028511	372	42496.55Da	5.87	41.4	85.51	-0.459
ClATG101a	EVM0067756	218	25469.17Da	6	31.31	83.99	-0.421
ClATG101b	EVM0080129	202	23477.84Da	5.99	33.69	82.43	-0.435
ClTORa	EVM0004734	2453	275769.36Da	6.54	43.8	100.42	-0.101
ClTORb	EVM0084899	2464	276458.09Da	6.55	42.62	100.17	-0.112
ClVPS15	EVM0060442	1430	160520.93Da	6.1	54.67	88.69	-0.203
ClVPS34	EVM0034556	781	89617.17Da	6.41	41.67	91.75	-0.308
ClVTI12a	EVM0017766	231	26178.29Da	9.92	55.17	99.61	-0.411
ClVTI12b	EVM0061062	190	21684.22Da	9.21	61.29	110.84	-0.298
ClVTI12c	EVM0078301	173	19559.27Da	5.89	53.87	86.88	-0.557
ClVTI12d	EVM0083460	220	24604.34Da	9.47	47.09	102.45	-0.4
