# Supplementary material for: Genome-Wide Identification of Autophagy-Related Gene Family and Gene Expression Analysis of the CmATG8 Under Heat Stress in Chrysanthemum
Source: Int J Mol Sci. 2025 Sep 5;26(17):8642. doi: 10.3390/ijms26178642 (PMC12428883; doi:10.3390/ijms26178642)
Supplement: Supplementary file 1 [file ijms-26-08642-s001.zip › Table S4.pdf]

Table S4. All primers used in this experiment

**Primer name**	**Sequence（5′-3′）**
qCmEF1α-R	CCATTCAAGCGACAGACTCA
qCmEF1α-F	TTTTGGTATCTGGTCCTGGAG
qCmATG8a-R	GTAACATACAAGAAACCATCCACATCC
qCmATG8a-F	GATAATGCCTTGCCACCCACAG
qCmATG8b-R	AGGGACCAGGTACTTCTTCTTATCG
qCmATG8b-F	CATGCCGAGGCTTCAAGGATTAG
qCmATG8c-R	AGTTGGTGGTAGCATGTTCTTGAC
qCmATG8c-F	AGTTCGTTTATGTGGTTCGTAAGAGG
qCmATG8d-R	CCTCTTTCTCACAACATACACAAACTG
qCmATG8d-F	AACCGACATACCTGACATTGACAAG
qCmATG8e-R	GGAACATCGCTTCTTTCTGCTTTC
qCmATG8e-F	TGCCGAGGCTGCTAGAATTAGAG
qCmATG8f-R	AATCCGTCATCATCCTTCTTCTCATC
qCmATG8f-F	GTAGGGCAATTTGTGTACGTGATTC
qCmATG8g-R	TCCTCTTTCTCACCACATACACAAAC
qCmATG8g-F	AACCGACATACCTGACATTGACAAG
qCmATG8h-R	ACATACAAGAAACCATCCTCATCCTTC
qCmATG8h-F	GATAATGCCTTGCCACCCACAG
qCmATG8i-R	ACATACAAGAAACCATCCTCATCCTTC
qCmATG8i-F	ACGCCTTGCCACCCACAG
qCmATG8j-R	CACCACTGTATGTCATGTAGAGGAAG
qCmATG8j-F	CCACCTACTGCTGCAATGATGTC
qCmATG8k-R	AGTTGGTGGTAGCATGTTCTTGAC
qCmATG8k-F	AGTTCGTTTATGTGGTTCGTAAGAGG
qCmATG8l-R	AGTTGGTGGTAGCATGTTCTTGAC
qCmATG8l-F	AGTTCGTTTATGTGGTTCGTAAGAGG
qCmATG8m-R	CGACAATCACCGGAATCCTATCAG
qCmATG8m-F	ATGGCAAAGAGTTTATTCAAGCAAGAG
qCmATG8n-R	CCTCTTTCTCACAACATACACAAACTG

qCmATG8n-F	AACCGACATACCTGACATTGACAAG
qCmATG8o-R	AGCAGTAGGTGGGAGGATGTTC
qCmATG8o-F	AGCGACATTCCTGACATTGACAAG
qCmATG8p-R	CACCACTGTATGTCATGTAGAGGAAG
qCmATG8p-F	CCACCTACTGCTGCAATGATGTC
qCmATG8q-R	GGAACATCGCTTCTTTCTGCTTTC
qCmATG8q-F	CGCTGAGGCTGCTAGAATTAGAG
